# Therapeutic mRNA delivery of CRISPR-Cas9 to the trabecular meshwork reverses ocular hypertension in myocilin glaucoma

**DOI:** 10.1016/j.omtm.2025.101614

**Published:** 2025-10-11

**Authors:** Sam Yacoub, Balasankara Reddy Kaipa, Linya Li, Sarahi Rios, Ramesh Kasetti, Prabhavathi Maddineni, Abbot F. Clark, Gulab S. Zode

**Affiliations:** 1 Department of Pharmacology and Neuroscience and North Texas Eye Research Institute, University of North Texas Health Science Center, Fort Worth, TX 76107, USA; 2 Gavin Herbert Eye Institute-Center for Translational Vision Research, Department of Ophthalmology, Department of Physiology and Biophysics, University of California, Irvine, Irvine School of Medicine, Irvine, CA 92697, USA; 3 Department of Ophthalmology, School of Medicine, University of Missouri, Columbia, MO 65212, USA

**Keywords:** glaucoma, trabecular meshwork, myocilin, juvenile-onset glaucoma, gene editing, protein misfolding, ocular hypertension, mouse model of glaucoma, mRNA delivery, Cas9, liposomes

## Abstract

Mutations in the myocilin gene (*MYOC*) are the leading genetic cause of primary open angle glaucoma (POAG), the most common glaucoma type. These mutations trigger a toxic gain-of-function phenotype, causing the misfolded MYOC protein to accumulate in the endoplasmic reticulum (ER) of trabecular meshwork (TM) cells, leading to ER stress, TM cell death, and elevation of intraocular pressure (IOP). Here, we demonstrate that the delivery of Cas9 mRNA via a cationic lipid polymer (lipoplex) targets the TM selectively and edits the *MYOC* gene, thereby rescuing a mouse model of glaucoma. A single intracameral injection of Cre-mRNA lipoplex resulted in mutant MYOC expression in the TM, leading to glaucoma in a recently developed Cre-inducible mouse model of glaucoma. Lipoplex encapsulating Cas9 and guide RNA targeting *MYOC* knocked out *MYOC*, reduced intracellular accumulation of mutant MYOC, and relieved ER stress, thereby rescuing a mouse model of *MYOC*-associated glaucoma. Our studies further establish the ocular safety and efficacy of non-viral Cas9-sgRNA delivery to the TM, offering a novel, one-time therapeutic strategy for inherited glaucoma caused by *MYOC* mutations.

## Graphical abstract

**Figure:**
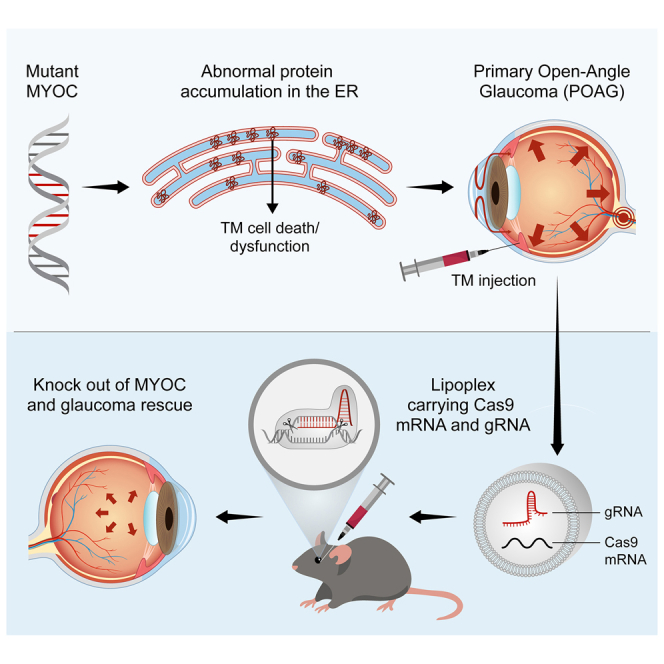

## Teaser

This study establishes proof of concept for delivering therapeutic genes to the trabecular meshwork using an mRNA-based approach. Non-viral CRISPR-Cas9 mRNA delivery edits mutant *MYOC*, alleviates ER stress, and reverses ocular hypertension in myocilin glaucoma, offering a safe, one-time therapeutic strategy beyond viral vectors.

## Introduction

Glaucoma is a group of optic neuropathies characterized by the gradual loss of retinal ganglion cells (RGCs) and degeneration of optic nerve axons, resulting in irreversible vision loss.^1^ Glaucoma is the second most prevalent blinding ocular condition worldwide. Glaucoma is predicted to affect more than 112 million people by 2040. Primary open-angle glaucoma (POAG) is the most prevalent form, accounting for 74% of the total glaucoma cases globally.^2^ Elevated intraocular pressure (IOP) is the leading risk factor and the only modifiable therapeutic target for treating POAG.^3^^,^^4^ Normal IOP relies on balancing the inflow and outflow of aqueous humor (AH). The trabecular meshwork (TM), a porous sieve-like tissue encircling the iridocorneal angle of the eye, is responsible for regulating aqueous humor outflow, and TM dysfunction increases outflow resistance, leading to elevated IOP.^5^^,^^6^ Many factors can cause TM damage, including aging, fibrosis, oxidative stress, inflammation, reduced phagocytosis, extracellular matrix deposition, decreased TM cell number, and genetic defects.^6^^,^^7^^,^^8^^,^^9^^,^^10^^,^^11^^,^^12^^,^^13^^,^^14^ However, the molecular pathogenic mechanisms underlying TM damage are not clear.

Despite the strong genetic basis of glaucoma, clearly defined causative genetic mutations have been identified in only a fraction of patients. Mutations in *MYOC* are the most common*,* well-established, and well-understood genetic causes of glaucoma.^15^^,^^16^^,^^17^ *MYOC* encodes a secreted protein, myocilin, the first and most comprehensively studied gene associated with hereditary forms of POAG.^16^^,^^18^^,^^19^^,^^20^^,^^21^ Many pathogenic *MYOC* mutations have been identified, most clustered in exon 3 encoding the olfactomedin domain.^16^ Approximately 4% of POAG cases and 30% to 40% of heritable juvenile glaucoma cases are attributed to mutations in the *MYOC* gene.^22^ Pathogenic mutations in the *MYOC* gene produce misfolded myocilin, which accumulates in the endoplasmic reticulum (ER), causing chronic ER stress and leading to the dysfunction or death of TM cells.^16^^,^^18^^,^^22^^,^^23^^,^^24^^,^^25^^,^^26^^,^^27^^,^^28^^,^^29^^,^^30^^,^^31^ Moreover, *MYOC*-associated juvenile open-angle glaucoma (JOAG) often exhibits reduced responsiveness to current medications because these treatments do not address the underlying molecular pathology caused by *MYOC* mutations.

*MYOC* is widely expressed in various normal tissues and organs,^32^^,^^33^ yet *MYOC*-associated diseases manifest exclusively in the eye. Among ocular tissues, *MYOC* expression is highest in the TM.^16^^,^^18^^,^^34^ *Myoc* knockout mice or humans with homozygous deletions of *MYOC* do not exhibit glaucoma phenotypes,^35^^,^^36^^,^^37^ indicating that wild-type *MYOC* is not required for IOP regulation. Studies using various knock-in and knockout mouse models suggest that *MYOC* mutations are gain-of-function mutations, in which the mutant MYOC protein accumulates in the ER, inducing ER stress and TM cell death.^16^^,^^18^^,^^26^^,^^27^^,^^31^ These events lead to increased outflow resistance and elevated IOP.^27^^,^^30^ Hence, the knockout of *MYOC* emerges as a promising therapeutic approach for the treatment of *MYOC*-related glaucoma.^38^

CRISPR-Cas has become the leading gene editing technology, initially discovered as an adaptive immune defense mechanism in bacteria.^39^ Unlike protein-DNA interactions, the CRISPR-Cas system utilizes RNA-DNA binding, where a guide RNA directs the Cas enzyme to specific DNA sequences for precise editing. This RNA-based targeting mechanism offers exceptional accuracy and versatility, establishing CRISPR-Cas as a powerful tool in genetic modification.^39^^,^^40^^,^^41^^,^^42^^,^^43^ Using viral vectors, our group has demonstrated successful gene editing of *MYOC* in mice and human donor eyes.^38^^,^^44^

While viral delivery of CRISPR components has demonstrated promising gene editing in the TM,^38^^,^^44^ it is associated with several disadvantages. One primary concern is the potential for inducing strong immune responses and inflammation in transduced tissues, particularly with commonly used vectors like adenovirus, which may reduce the efficacy of gene editing.^38^ Additionally, there is a risk of insertional mutagenesis with some viral vectors, where the viral DNA integrates into the host genome at unintended sites, potentially disrupting essential genes or regulatory elements.^45^^,^^46^^,^^47^ Furthermore, viral vector-mediated sustained Cas9 expression can increase off-target effects, as prolonged Cas9 activity may inadvertently edit DNA sequences that are similar, but not identical, to the intended target, potentially leading to unintended genetic alterations. We observed significant off-target effects when Cas9 was delivered to the TM using lentiviral vectors.^44^ Moreover, immune responses against the viral vectors themselves can limit the effectiveness of the delivery system, and the size limitations of viral vectors may restrict the size of the genetic payload that can be delivered.^48^^,^^49^ Finally, the production and purification of viral vectors can be complex and costly, limiting their scalability and accessibility for widespread therapeutic applications.

In contrast, non-viral delivery systems for gene editing tools are rapidly advancing, providing safer and more efficient alternatives to viral vectors.^50^^,^^51^ These include lipid nanoparticles, electroporation, and lipoplexes/polyplexes, which generally rely on electrostatic interactions to facilitate the efficient delivery of CRISPR components without the risks associated with viral genome integration.^51^^,^^52^^,^^53^ Among these, the use of mRNA to deliver CRISPR components is gaining prominence due to its transient expression and reduced risk of genomic integration.^54^^,^^55^^,^^56^^,^^57^ In this study, we utilized a commercially available cationic lipid polymer (In Vivo Jet Messenger from Polyplus), which forms a lipoplex with mRNA components to deliver therapeutics to the TM. We first evaluated whether Cre-mRNA lipoplex induces mutant *MYOC* selectively in the TM, leading to glaucoma in a recently developed Cre-inducible mouse model of *MYOC*-glaucoma. Next, we investigated whether liposomes carrying Cas9 and guide RNA edit *MYOC* and reduce elevated IOP in this mouse model of *MYOC*-associated glaucoma.

## Results

### Cre-mRNA lipoplex exhibits specific tropism to TM cells *in vitro* and *in vivo*

**Figure 1:**
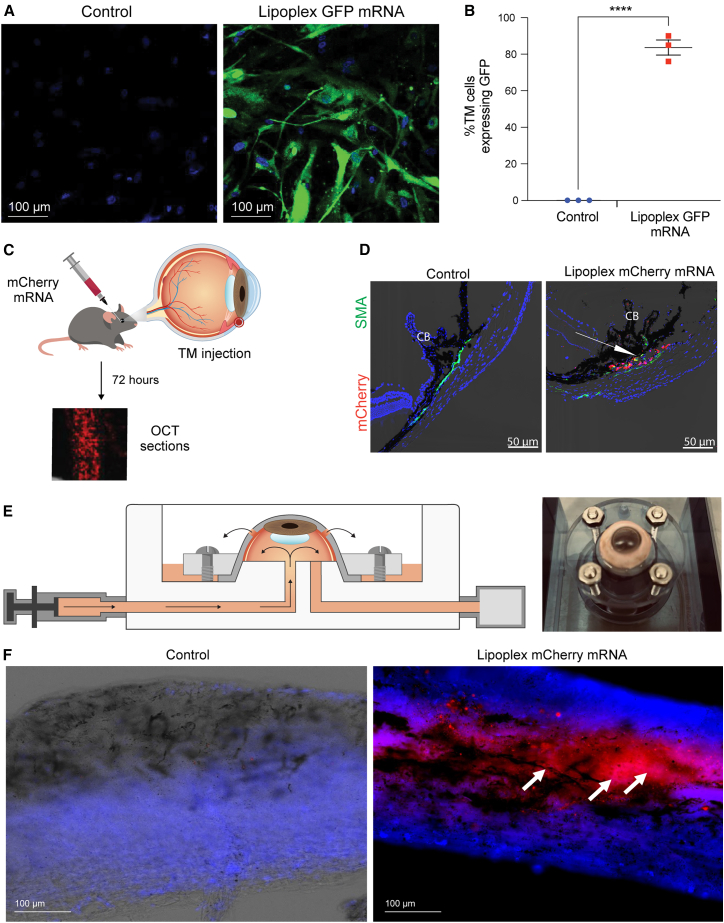
Cre-mRNA lipoplex exhibits selective tropism to TM cells *in vitro* and *in vivo* (A) Human primary TM cells were incubated with lipoplex GFP-mRNA (0.5 μg/mL). Twenty-four hours after incubation, robust GFP expression was observed in most TM cells, indicating successful mRNA delivery and translation into a functional protein. (B) Quantitative analysis of GFP-positive cells over the total number of TM demonstrates that 82% of TM cells produced GFP protein after treatment with lipoplex GFP mRNA. (Human primary TM and GTM3 cells, *N* = 3, *p* < 0.00001). (C) A schematic representation of the study design. C57BL/6J mice received an intracameral injection of lipoplex mCherry (1 μg/eye). Cross-sectional imaging of the anterior segment was acquired three days post-injection. (D) Anterior segment cross-sections were immunostained with α-smooth muscle actin (α-SMA, green) to identify TM cells. Confocal microscopy revealed mCherry protein expression (red) in the TM region marked by α-SMA in mCherry mRNA-injected mice compared with controls. *N* = 4. (E) A schematic representation of an *ex vivo* perfusion organ culture (POC) system to study the TM outflow pathway. (F) Anterior segments from donor eyes were perfusion-cultured, and one eye received an intracameral injection of lipoplex mCherry (1 to 5 μg/eye) while the contralateral eye received lipoplex buffer. TM tissue was isolated and flat-mounted on a glass slide with DAPI. Fluorescence imaging of dsRed protein in the TM of lipoplex control and lipoplex mCherry mRNA-treated eyes is shown (*n* = 3). Arrows indicate the TM.

We first investigated whether lipoplex formulations can deliver mRNA to TM cells and whether TM cells can produce functional protein *in vitro* and *in vivo*. A GFP-mRNA lipoplex was formulated using the cationic lipid polymer PolyPlus JetMessenger, as described in the materials and methods. Human primary TM cells were incubated with GFP-mRNA lipoplex (0.5 μg/mL) for 24 h. We observed a robust expression of GFP protein 24 h post-transfection, with almost 82% of TM cells expressing the GFP protein (Figures 1A and 1B). Next, we determined whether mRNA-Cre exhibits functional activity *in vitro* by incubating mouse fibroblasts obtained from *Tg.CreMYOC*^*Y437H*^ mice. Mutant *MYOC* fused with dsRed is not expressed under normal conditions. Upon Cre introduction, fibroblasts induce dsRed-tagged mutant *MYOC*. Cre-inducible skin fibroblasts were incubated with 0.5 μg/mL Cre-mRNA lipoplex for 24 h, and dsRed was examined via confocal microscopy. Fibroblasts treated with liposome-Cre mRNA induced DsRed-MYOC, indicating successful Cre activity. Approximately 90% of the treated fibroblasts exhibited dsRed expression within 24 h when transfected with Cre-mRNA lipoplex compared to cells that received JetMessenger alone (Figure S1). We next examined whether mRNA-mCherry lipoplex can produce mCherry protein in the mouse TM *in vivo*. mCherry mRNA lipoplex (1 μg/eye) was injected intracamerally in C57BL/6J mice. Expression of mCherry was visualized after 3 days of treatment (Figures 1C and 1D; Figure S1). To anatomically delineate the TM region, we used α-smooth muscle actin (α-SMA) staining. While α-SMA is not specific to the TM and also labels ciliary muscle cells,^58^^,^^59^ it provides a useful reference for identifying the TM region in mouse anterior segments. Sections were co-stained with α-SMA, and a robust mCherry signal was observed within the TM region, supporting that lipoplex-carrying mRNA can efficiently transduce TM cells *in vivo*. We also delivered mCherry mRNA via intravitreal injection with very similar results (i.e., selective expression in the TM; data not shown). Next, we explored whether mCherry mRNA lipoplex targets human TM tissue using a perfusion-cultured system (Figure 1E). A schematic representation of the perfusion setup, along with an example of the human anterior segment, demonstrates a successful perfusion system (Figure 1E). Anterior segments from donor eyes were perfused and injected intracamerally with lipoplex loaded with mCherry mRNA (5 μg). TM tissue was isolated after 3 days, and mCherry protein was visualized (Figure 1F). A robust mCherry protein was observed in the entire TM tissue, indicating strong tropism of mRNA to the TM. These data indicate that lipoplexes carrying mRNA exhibit a robust and selective tropism for mouse and human TM *in vitro* and *in vivo*.

### A single intracameral injection of Cre-mRNA lipoplex induces mutant *MYOC* selectively in TM cells and develops glaucoma in *Tg.CreMYOC*^*Y437H*^ mice

We have recently developed a Cre-inducible mouse model of *MYOC*-associated POAG (*Tg-CreMYOC*^*Y437H*^ mice). Mutant *MYOC* is tagged with dsRed, which is not expressed under normal conditions. Upon Cre introduction, mutant MYOC-DsRed is expressed in a tissue-specific manner. Previously, we have shown that helper adenovirus (HAd) 5-Cre induces mutant MYOC in the TM, elevating IOP and leading to glaucoma in *Tg-CreMYOC*^*Y437H*^ mice.^30^ Here, we explored whether lipoplex encapsulating Cre-mRNA can induce mutant *MYOC* in mouse TM and develop glaucoma in *Tg-CreMYOC*^*Y437H*^ mice.

### Induction of mutant *MYOC* in the TM

**Figure 2:**
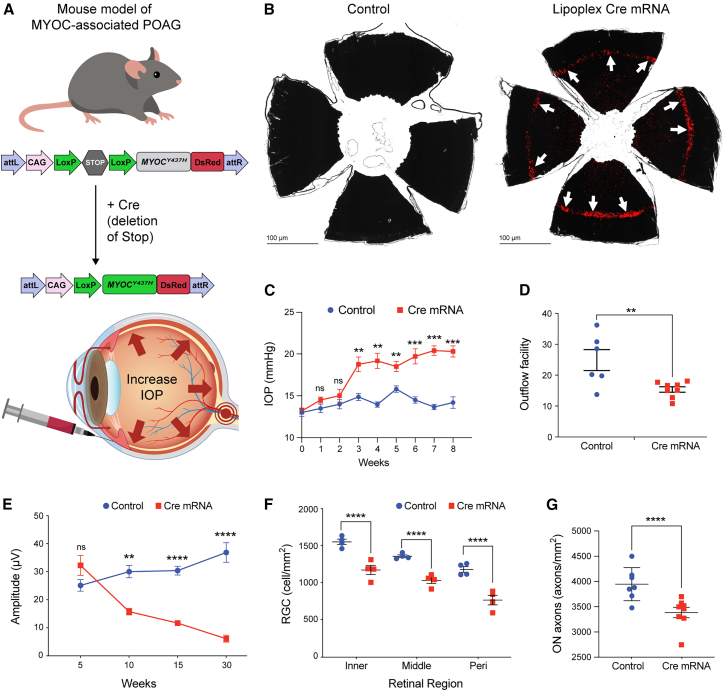
A single intracameral injection of Cre-mRNA lipoplex induces mutant *MYOC* selectively in TM cells, developing glaucoma phenotypes in *Tg.CreMYOC*^*Y437H*^ mice (A) A schematic representation of the study design. *Tg.CreMYOC*^*Y437H*^ mice harbor DsRed-tagged human *MYOC* gene carrying the Y437H mutation under the control of the Cre-lox system. An STOP cassette was placed upstream of the mutant *MYOC*-DsRed fusion gene to control expression, preventing its transcription under basal conditions. Upon introduction of Cre recombinase, the stop cassette is excised, enabling expression of the mutant MYOC-DsRed fusion protein specifically in Cre-expressing (targeted) cells. (B) Intracameral injection of lipoplex-Cre mRNA (1 μg/eye) was performed in *Tg.CreMYOC*^*Y437H*^ mice. DsRed-tagged mutant *MYOC* expression was selectively observed in the entire TM one week after injection via flat-mount of the anterior segment. Arrows point to the location of the TM. (C) Weekly IOP measurements revealed a significant and sustained elevation in IOP in Cre-injected *Tg.CreMYOC*^*Y437H*^ mice compared to those injected with lipoplex buffer (*n* = 22 Cre; *n* = 10 control). Data were analyzed using two-way ANOVA with multiple comparisons (∗∗∗*p* < 0.001). (D) The outflow facility was significantly reduced in *Tg.CreMYOC*^*Y437H*^ mice 20 weeks after Cre injection (*n* = 8) compared to mice injected with buffer (*n* = 6, ∗∗*p* < 0.01). (E) pERG was performed at 5, 10, 15, and 30 weeks post-Cre-injection to assess the function of RGCs. PERG analysis demonstrates significantly reduced pERG amplitude starting from 10 weeks of Cre injection (*n* = 8 Cre; *n* = 6 control, ∗∗*p* < 0.01, ∗∗∗*p* < 0.001). (F) RGC loss was further assessed by whole-mount retinal staining with the RBPMS antibody, revealing a significant ∼30% reduction in RGCs after 30 weeks of Cre-injection in *Tg.CreMYOC*^*Y437H*^ mice compared to controls (*n* = 4, ∗∗∗∗*p* < 0.0001). (G) Mean optic nerve (ON) axon counts revealed a significant loss in Cre-induced *Tg.CreMYOC*^*Y437H*^ mice, with a 43% reduction at 20 weeks post-Cre-injection (*n* = 7, ∗∗∗∗*p* < 0.0001).

A single intracameral injection of Cre-mRNA (1 μg/eye) formulated with in-vivo-jetRNA was performed in 3-month-old *Tg.CreMYOC*^*Y437H*^ mice (Figure 2A). Three days later, dsRed expression was examined using whole-mount anterior segments (Figure 2B) and cross-sections (Figure S2), demonstrating dsRed and mutant *MYOC* expression in the TM. Moreover, the entire TM exhibited robust dsRed expression after treatment with Cre-mRNA lipoplex, suggesting strong tropism of the lipoplex carrying Cre mRNA to the TM (Figure 2B). This finding was confirmed by the presence of mutant MYOC using western blot (Figure S2A) and anterior segment cross-section, which demonstrated a significant increase in dsRed protein in the TM (Figures S2B and S2C). We compared intracameral and intravitreal injections of liposome-mCherry mRNA and found that both delivery routes induced DsRed-MYOC expression in the TM (Figure S2B). Although sporadic dsRed expression was observed in the corneal endothelium following intracameral delivery, this route produced more consistent and uniform TM expression compared to intravitreal injection and was therefore chosen for subsequent studies.

### IOP elevation and outflow facility

Next, we investigated whether the selective expression of mutant *MYOC* in TM cells leads to IOP elevation in *Tg.CreMYOC*^*Y437H*^ mice. 4-month-old *Tg.CreMYOC*^*Y437H*^ mice were bilaterally injected intracamerally with either Cre-mRNA (1 μg/eye) lipoplex (Treated) or lipoplex buffer (Control). IOPs were monitored weekly. Starting from 3 weeks of injection, Cre-injected *Tg.CreMYOC*^*Y437H*^ mice demonstrated a significant and persistent increase in IOPs compared to those treated with the vehicle alone (Figure 2C). Ocular hypertension (OHT) in cre-mRNA-injected eyes over control eyes was 3.89 mmHg higher and remained elevated throughout the course of this study. We next examined whether IOP elevation is due to reduced outflow facility through the TM by measuring the outflow facility (Figure 2D). Outflow facility measurement demonstrated a significant reduction in outflow facility in *Tg.CreMYOC*^*Y437H*^ mice after 20 weeks of Cre-injection, indicating that mutant *MYOC* expression in the mouse TM causes TM dysfunction, resulting in reduced outflow facility and IOP elevation.

### RGC loss

To examine whether Cre-induced OHT leads to functional loss of RGCs, we performed pERG after 5, 10, and 15 weeks of injection in *Tg.CreMYOC*^*Y437H*^ mice. Cre-mRNA-injected eyes showed a significant reduction in pERG amplitudes starting from 10 weeks of Cre-injection in *Tg.CreMYOC*^*Y437H*^ mice (Figure 2E; Figures S3A and S3B). Significantly increased pERG latency was observed from 15 weeks of Cre-injection in *Tg.CreMYOC*^*Y437H*^ mice. These data indicate that sustained OHT leads to significant and progressive functional loss of RGCs in *Tg.CreMYOC*^*Y437H*^ mice. Whole-mount retina staining with the RGC-specific marker RBPMS was performed in 20 weeks of Cre-injection in *Tg.CreMYOC*^*Y437H*^ mice. The total number of RGCs was analyzed from the whole-mount retina in a masked and automated manner using ImageJ software. Cre-injected eyes showed significant RGC loss compared to the Veh-injected *Tg.CreMYOC*^*Y437H*^ eyes. There was a ∼30% RGC loss in Cre-injected eyes compared to Veh-injected eyes (Figure 2F; Figure S3C).

### Optic nerve degeneration

Optic nerve degeneration is a hallmark feature of human glaucoma and a critical pathological event in glaucomatous neurodegeneration, leading to vision loss.^60^^,^^61^ To investigate whether sustained OHT leads to optic nerve degeneration in *Tg.CreMYOC*^*Y437H*^ mice, we performed PPD staining of optic nerve cross-sections at 20 weeks after injection with vehicle or Cre-mRNA (Figures S4A and S4B). We observed a significant and notable optic nerve degeneration in Cre-injected *Tg.CreMYOC*^*Y437H*^ mice, as evidenced by darkly stained axons, vacuoles, active gliosis, and the formation of glial scars. Cre-mRNA-injected eyes exhibited a significant 43% reduction in optic nerve axons (Figure 2G). These data indicate that a single intracameral injection of Cre-mRNA lipoplex induces mutant MYOC in the TM, causing TM dysfunction and IOP elevation in *Tg.CreMYOC*^*Y437H*^ mice. Sustained IOP elevation leads to progressive RGC loss and optic nerve degeneration in *Tg.CreMYOC*^*Y437H*^ mice.

### Lipoplex encapsulating Cas9-mRNA and gRNA targeting *MYOC* knocks out MYOC in cultured TM cells

**Figure 3:**
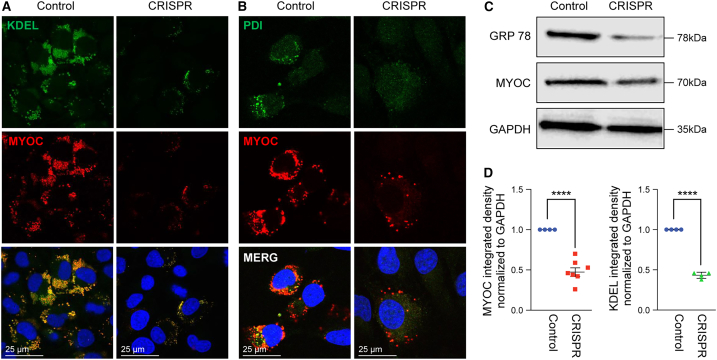
Lipoplex encapsulating Cas9-mRNA and gRNA targeting *MYOC* reduces intracellular accumulation of mutant myocilin protein in cultured TM cells GTM3 cells stably expressing mutant *MYOC* were transfected with lipoplexes containing either Cas9 mRNA + scrambled gRNA (Control) or Cas9 mRNA + *MYOC*-targeting gRNA (Treated). Immunostaining for (A) KDEL or (B) Protein disulfide isomerase (PDI) revealed a reduction in intracellular accumulation of mutant myocilin protein in the ER and its associated ER stress in treated cells compared to controls. *N* = 3. (C) Western blot analysis of MYOC and GRP78 in cell lysates from TM cells expressing mutant MYOC treated with either Cas9 mRNA + scrambled gRNA (Control) or Cas9 mRNA + *MYOC*-targeting gRNA (Treated). (D) Densitometric analysis of the Western blots revealed a significant reduction in MYOC protein levels in CRISPR-Cas9-treated cells compared to controls. GRP78, an ER stress marker, was reduced significantly upon Cas9 mRNA + *MYOC*-targeting gRNA treatment compared to controls, (*n* = 7 MYOC, *n* = 4 KDEL, ∗∗∗∗*p* < 0.0001).

In previous studies, we successfully utilized viral delivery of the CRISPR-Cas9 system to achieve knockout of the mutant *MYOC* gene.^34^^,^^35^ In the present study, we explored whether Cas9-mRNA and gRNA knock out *MYOC in vitro*. GTM3 cells stably expressing DsRed-tagged human *MYOC* with either the Y437H or G364V mutations were utilized. These mutations impair MYOC secretion, leading to its intracellular accumulation.^14^^,^^23^^,^^56^^,^^57^^,^^58^ We first determined whether the Cas9 protein is produced after transfection with Cas9 mRNA. Western blot analysis of GTM3 cells incubated with Cas9 mRNA for 0–72 h demonstrated detectable Cas9 protein expression between 6 and 24 h, with maximum expression at 9 h (Figure S5). Next, GTM3 cells stably expressing mutant *MYOC* were incubated with lipoplex containing Cas9mRNA+scrambled gRNA (Control) or Cas9mRNA+gRNA targeting *MYOC* (Treated). MYOC and its intracellular accumulation in the ER were examined via immunostaining. A reduction in intracellular dsRed was observed in GTM3 cells treated with Cas9 mRNA and g*MYOC* compared to cells transfected with the control (Figures 3A and 3B). We also observed a reduction in the colocalization of MYOC with the ER marker KDEL, indicating a decrease in the intracellular accumulation of MYOC in GTM3 cells treated with Cas9 and *gMYOC*. The effect of Cas9 mRNA on myocilin knockdown was determined via western blot analysis (Figure 3C). Densitometric analysis of the western blot showed a significant reduction in *MYOC* in Cas9-treated cells compared to controls. Quantification of WB of MYOC and ER stress marker, GRP78 revealed a 53% and 51% reduction in MYOC and GRP78 levels following Cas9/gRNA treatment (Figure 3D). These data establish that Cas9 mRNA and g*MYOC* lipoplex reduce intracellular MYOC protein accumulation in the ER of TM cells.

### Cas9 mRNA and g*MYOC* lipoplex reduce mutant MYOC in the TM, decreasing elevated IOP in *Tg.CreMYOC*^*Y437H*^ mice

**Figure 4:**
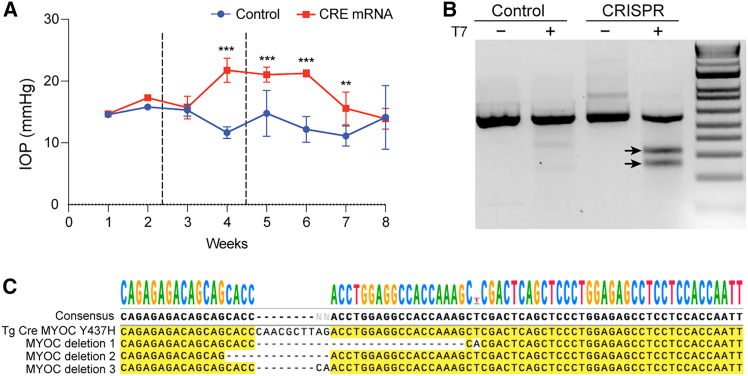
Lipoplex carrying Cas9 mRNA and gRNA targeting *MYOC* reduces elevated IOP in *Tg.CreMYOC*^*Y437H*^ mice (A) IOP measurements in *Tg-MYOC*^*Y437H*^ mice. *Tg.CreMYOC*^*Y437H*^ mice were first injected with lipoplex buffer (Control) or Cre mRNA (1^st^ vertical dashed line), and IOP was measured weekly. Cre mRNA injected *Tg.CreMYOC*^*Y437H*^ mice developed a significant IOP elevation 4 weeks post-Cre injection. At this stage, ocular hypertensive *Tg.CreMYOC*^*Y437H*^ mice were injected intracamerally with lipoplex encapsulating Cas9mRNA+gRNA targeting *MYOC* (2^nd^ vertical dashed line). IOPs were measured weekly (*n* = 6 each group). CRISPR treatment reduced elevated IOP significantly back to the baseline. Two-way ANOVA with repeated measurements and Bonferroni post-hoc analysis were performed. Data represented as mean ± SEM; ∗∗*p* < 0.01, and ∗∗∗*p* < 0.001. (B) T7E1 assay demonstrated a cleaved product in DNA samples from GTM3 cells treated with Cas9 mRNA and gRNA targeting *MYOC* (black arrows). No cleaved product was observed in the scrambled gRNA-treated DNA samples (Control). (C) Ocular hypertensive *Tg.CreMYOC*^*Y437H*^ mice were injected intracamerally with either lipoplex encapsulating Cas9mRNA+scrambled gRNA (Control) or Cas9mRNA+gRNA targeting *MYOC* (Treated). Next-generation sequencing was performed on genomic DNA, which revealed multiple deletions in *MYOC* exon 1, indicating successful knockout in Cas9 mRNA + g*MYOC*-injected mice compared to scrambled gRNA controls (*n* = 3). Note: “*MYOC* deletion 1–3” refers to deletions observed in each of the three mice examined.

**Figure 5:**
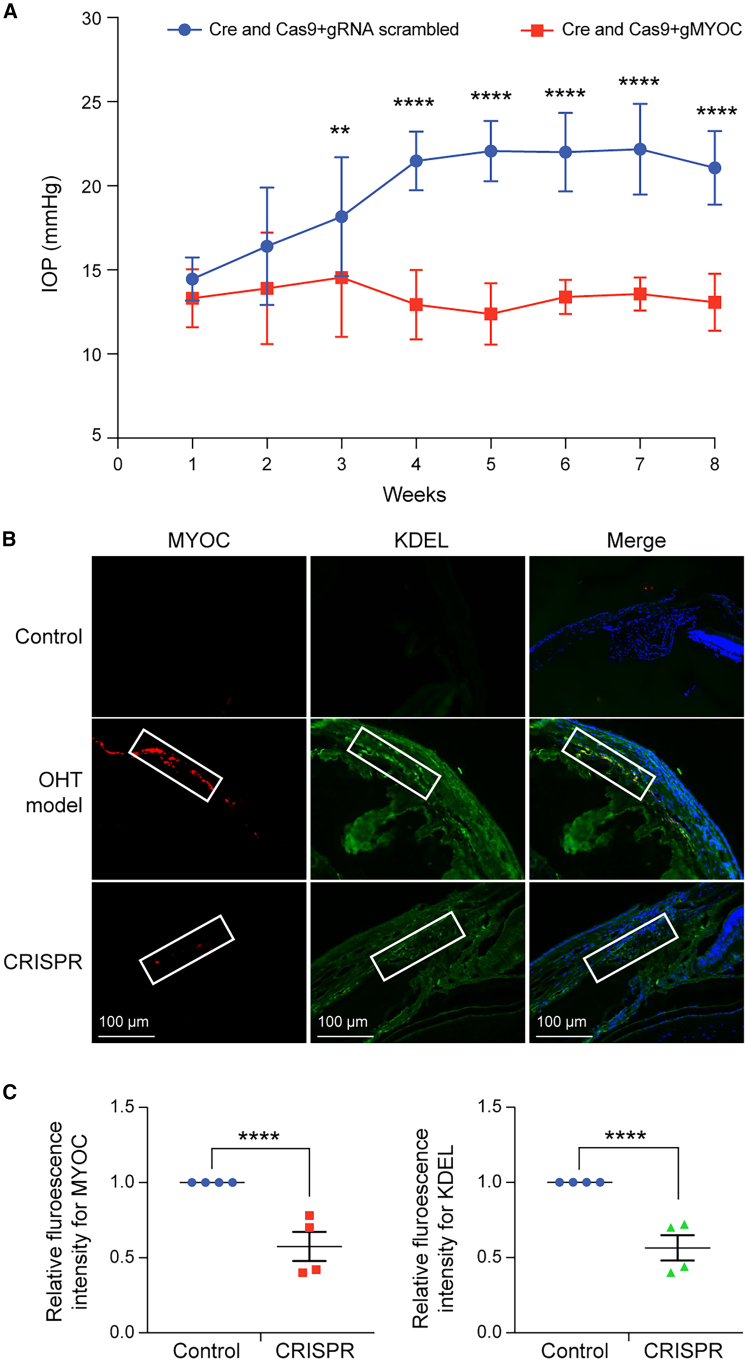
Cas9 mRNA and gRNA targeting *MYOC* rescues the glaucomatous pathology induced by mutant MYOC in the TM of *Tg.CreMYOC*^*Y437H*^ mice (A) *Tg.CreMYOC*^*Y437H*^ mice were injected with lipoplex-Cre mRNA along with Cas9 mRNA+scrambled gRNA or gRNA targeting *MYOC*. IOP was measured weekly. *Tg.CreMYOC*^*Y437H*^ mice injected with Cre and Cas9+scrambled gRNA developed a significant IOP elevation 3 weeks post-injection and sustained IOP elevation throughout the study. *Tg.CreMYOC*^*Y437H*^ mice injected with lipoplex loaded with Cre and Cas9 mRNA+gRNA targeting *MYOC* did not show changes in IOPs, indicating rescue of ocular hypertension in *Tg.CreMYOC*^*Y437H*^ mice. Two-way ANOVA with repeated measurements and Bonferroni post-hoc analysis were performed. Data represented as mean ± SEM; ∗∗*p* < 0.01, and ∗∗∗*p* < 0.001. Representative immunostaining for MYOC and KDEL (B) and its quantification (C) demonstrated that Cas9 mRNA+g*MYOC* significantly reduces MYOC and its co-localization with the ER marker KDEL in the TM of *Tg.CreMYOC*^*Y437H*^ mice compared to control-treated mice. Quantification revealed a 54% reduction in MYOC levels and a 55% reduction in KDEL levels following Cas9/gRNA treatment (*n* = 4, ∗∗∗∗*p* < 0.0001).

We explored whether Cas9 mRNA and gMYOC lipoplex edits *MYOC*, reducing its intracellular accumulation and elevated IOP in *Tg.CreMYOC*^*Y437H*^ mice. Lipoplex encapsulating Cas9 and gRNA was formulated using the *in vivo* jet messenger cationic polymer, and analysis of the formulation revealed that the lipoplex exhibits a uniform particle size (Figure S6). A single intracameral injection of Cre-mRNA (1 μg/eye) was performed in 3-month-old *Tg.CreMYOC*^*Y437H*^ mice. IOPs were monitored weekly to ensure IOP elevation. Cre-mRNA induced significant IOP elevation after 4 weeks of injection in *Tg.CreMYOC*^*Y437H*^ mice. After 5 weeks of Cre-injection, ocular hypertensive *Tg.CreMYOC*^*Y437H*^ mice were divided randomly into two groups. One group was injected intracamerally with lipoplex loaded with Cas9mRNA+scrambled gRNA (Control), while the other was injected with Cas9mRNA+gRNA targeting *MYOC* (Treated). It should be noted that there were no differences in RGC counts at the initiation of CRISPR intervention (5 weeks after Cre transfection of the TM). IOP measurements revealed that Cas9 mRNA and gRNA *MYOC* lipoplex reduced IOP significantly within 3 weeks of injection compared to the control group (Figure 4A). To confirm *MYOC* editing, we further performed T7 endonuclease (T7E1) assay, which demonstrated a cleaved product in DNA samples from GTM3 cells treated with Cas9 mRNA and g*MYOC* (Figure 4B). Based on our T7E1 analysis, we found approximately 18% MYOC editing efficiency (Figure S7). No cleaved product was observed in the untreated control and scrambled gRNA-treated DNA samples. To precisely define the editing outcomes *in vivo*, we performed next-generation sequencing of the MYOC locus using pooled iridocorneal angle tissues from three Cas9 mRNA + gMYOC-treated *Tg.CreMYOC*^*Y437H*^ mice (Figure 4C). Compared to Cas9 mRNA+scrambled gRNA-injected *Tg.CreMYOC*^*Y437H*^ mice, Cas9 mRNA+g*MYOC*-injected *Tg.CreMYOC*^*Y437H*^ mice demonstrated multiple deletions in exon 1 of *MYOC*, resulting in a knockout of *MYOC*. Next, we explored whether the lipoplex of Cas9 mRNA and g*MYOC* prevents IOP elevation in *Tg.CreMYOC*^*Y437H*^ mice by administering Cre mRNA and Cas9+gMYOC at the beginning of the study (Figure 5A). *Tg.CreMYOC*^*Y437H*^ mice were injected with Cre mRNA along with Cas9+gMYOC or scrambled RNA and IOPs were monitored weekly. While Cre mRNA and Cas9+scrambled RNA injected *Tg.CreMYOC*^*Y437H*^ mice developed significant ocular hypertension 3-weeks post injection, *Tg.CreMYOC*^*Y437H*^ mice injected with Cre and Cas9+gMYOC did not elevate IOP significantly, and IOPs in these mice remained normal throughout the study. These data indicate that *MYOC* editing prevents ocular hypertension in *Tg.CreMYOC*^*Y437H*^ mice.

We next investigated whether the lipoplex of Cas9+gMYOC prevents the intracellular accumulation of mutant MYOC in the TM of the above *Tg.CreMYOC*^*Y437H*^ mice. Immunostaining confirmed that Cas9 treatment reduced MYOC and its co-localization with ER marker, KDEL, in the TM of *Tg.CreMYOC*^*Y437H*^ mice compared to control-treated mice (Figure 5B). Quantification of MYOC and KDEL using ImageJ revealed a 54% and 55% reduction in MYOC and KDEL levels following CRISPR-Cas9/gRNA treatment (Figure 5C). These data indicate that lipoplex containing Cas9 mRNA and g*MYOC* edits *MYOC*, reduces its intracellular accumulation, and prevents ocular hypertension in *Tg.CreMYOC*^*Y437H*^ mice. Although we did not appear to get a complete knockout of MYOC in the TM tissues of these mice (Figure 4B), we decreased the mutant MYOC burden sufficiently to both intervene (Figure 4A) and prevent (Figure 5A) mutant MYOC-induced ocular hypertension in mice.

### Cas9 mRNA delivery demonstrates excellent ocular safety and minimal off-target effects *in vivo*

**Figure 6:**
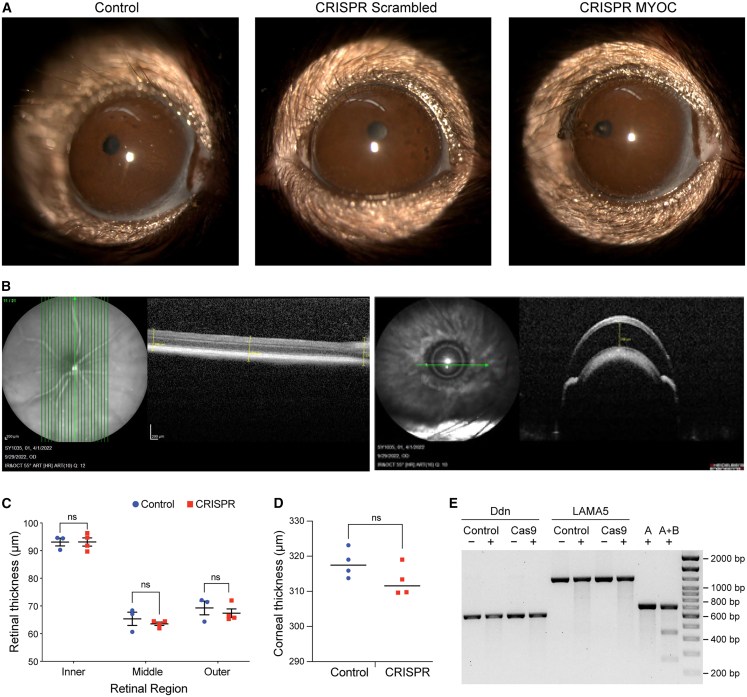
The ocular safety of lipoplex carrying Cas9 mRNA in *Tg.CreMYOC*^*Y437H*^ mice (A) Representative slit-lamp images of mouse eyes from control and Cas9 mRNA with scrambled or *MYOC* gRNA injected *Tg.CreMYOC*^*Y437H*^ mice. *N* = 3. (B) Representative OCT imaging showing that no obvious ocular inflammation is associated with either CRISPR mRNA scrambled or CRISPR mRNA MYOC mice (*N* = 4). (C) No significant differences in retinal thickness or (D) corneal thickness between Cas9 mRNA + gMYOC or Cas9 + scrambled gRNA were observed. (E) T7 endonuclease assays were performed on the top two predicted off-target sites, Lama5 and Ddn. While the positive control exhibited the expected cleavage, no cleavage products were observed at the predicted off-target sites.

Ensuring the safety of gene-editing technologies is critical for their successful translation into clinical applications. In our previous studies, viral vector-mediated delivery of Cas9 produced significant off-target effects.^44^ In the present work, we investigated whether lipoplex-mediated delivery of Cas9 mRNA and guide RNA (gRNA) targeting *MYOC* enhances ocular safety and minimizes off-target effects by enabling transient Cas9 expression. Adult *Tg.CreMYOC*^*Y437H*^ mice were injected with lipoplex buffer or lipoplex carrying Cas9+scrambled gRNA and subjected to live optical coherence tomography (OCT) imaging 10 weeks after injection (Figures 6A–6C). Anterior segment OCT images showed no evidence of ocular inflammation in Cas9 lipoplex-injected eyes compared to controls (Figure 6A), with the iridocorneal angle remaining open and corneal thickness comparable between groups. Posterior segment OCT imaging revealed no alterations in the retinal architecture or retinal thickness (Figure 6B) of Cas9-g*MYOC*-injected eyes. These findings were further corroborated by hematoxylin and eosin (H&E) staining of the anterior segment, which revealed no evidence of ocular inflammation, with ocular structures appearing similar between control and Cas9-injected eyes (Figure S8). Together, these findings establish the ocular safety of lipoplex-mediated Cas9 delivery. Previously, we observed significant off-target effects when Cas9 was delivered via lentiviral particles to mouse TM.^44^ Here, we examined whether lipoplex carrying Cas9 exhibited any off-target effects in the mouse TM of Cas9+g*MYOC*-treated *Tg.CreMYOC*^*Y437H*^ mice. NGS data confirmed on-target editing, with multiple deletions in exon 1 of Cas9+g*MYOC*-treated eyes (Figure 4C). *In silico* analysis by Cas9 finder revealed a low likelihood of off-target activity (Figure S9). To experimentally validate these findings, we performed *T7 endonuclease* assays on the top two predicted off-target sites, *Lama5* and *Ddn*. While the positive control exhibited the expected cleavage, no cleavage products were observed at the predicted off-target sites, suggesting high specificity of the *gRNA* for the *MYOC* gene (Figure 6E). These findings indicate that lipoplex delivery of Cas9 and g*MYOC* does not induce ocular toxicity or any detectable off-target activity *in vivo*.

## Discussion

In this study, we employed a lipoplex-based approach to deliver Cas9 mRNA to TM targeting *MYOC* in a POAG mouse model. Our findings provide a critical proof of concept demonstrating the feasibility of mRNA delivery to the TM using non-viral methods. Lipoplex-mediated mRNA exhibited a robust and selective tropism for the TM *in vitro*, *in vivo*, and in an *ex vivo* perfusion-cultured system. Specifically, we leveraged a lipoplex-mediated Cre mRNA delivery in our recently developed Cre-inducible *Tg.CreMYOC*^*Y437H*^ mouse model to induce the expression of mutant *MYOC* in the TM. Lipoplex carrying Cre mRNA produced mutant MYOC in the TM, elevating IOP and leading to the development of glaucoma in *Tg.CreMYO*^*CY437H*^ mice. We further employed lipoplex carrying Cas9 mRNA and g*MYOC* to knock out mutant *MYOC* in these mice, significantly reducing the accumulation of the mutant MYOC protein within the TM, thereby alleviating ER stress and lowering IOP. This strategy represents a promising advance toward developing a safe therapeutic intervention for *MYOC*-associated POAG, offering a means of addressing the underlying genetic etiology of the disease.

A key highlight of this study is the use of lipoplex-based mRNA as a non-viral delivery vehicle for CRISPR-Cas9, representing a significant advancement over conventional viral approaches. Our group previously utilized viral vectors, particularly adenovirus 5, AAV2 and lentiviral particles.^38^^,^^44^ However, these systems come with substantial risks^,^ including insertional mutagenesis, prolonged Cas9 expression leading to off-target effects, and immune responses that can limit repeated administration.^51^^,^^52^^,^^62^^,^^63^ Our previous study observed significant off-target effects using lentiviral vectors to express Cas9, likely due to sustained Cas9 production.^44^ Utilizing mRNA delivery to produce Cas9 transiently mitigates these concerns, offering a safer and more versatile alternative. We demonstrate that Cas9 is produced within hours of treatment and subsequently degraded, which can reduce off-target effects while enhancing TM-specific on-targeting.

With the added benefit of rapid, cost-effective synthesis, our lipoplex-based mRNA delivery approach opens new avenues for non-viral, repeatable, and precise gene-editing therapies for ocular diseases. The success of mRNA-based therapeutics in developing COVID-19 vaccines has demonstrated their potential for safe and effective gene delivery in humans.^64^ This proves their translational potential and further highlights their suitability for gene editing therapies, including those targeting ocular diseases.^65^

A novel approach to induce mutant MYOC using lipoplex-carrying Cre mRNA has produced robust glaucoma phenotypes in our recently developed *Tg.CreMYOC*^*Y437H*^ mice.^30^ Importantly, Cre-induced *Tg.CreMYOC*^*Y437H*^ mice closely mirror essential features of human *MYOC*-associated glaucoma, including IOP elevation due to TM dysfunction and glaucomatous neurodegeneration due to sustained IOP elevation. The Cre/*loxP* system, a widely utilized genetic tool that enables precise control over gene expression in specific tissues or at designated times,^66^ enabled us to specifically target and express the mutant *MYOC* gene in the TM, the primary disease site in *MYOC*-associated glaucoma. The lack of a TM-specific Cre mouse line has limited the use of the Cre-lox system, and our approach to induce Cre via mRNA provides an attractive strategy to induce Cre selectively in TM tissue to explore molecular mechanisms of other potential glaucoma-related genes using the Cre-lox system. Another major highlight of the lipoplex mRNA delivery system is its selectivity for the TM. Using mRNA expressing mCherry or Cre, our studies clearly show selective expression of the mCherry protein and functional activity of Cre in mouse TM. We compared intracameral and intravitreal injections of liposome-mCherry mRNA and found that both approaches induced dsRed-MYOC expression in the TM (Figure S2B). However, intracameral injection produced more consistent and uniform expression in the TM and, therefore, was selected for subsequent studies. We acknowledge that α-SMA is not a TM-specific marker, as it also labels ciliary muscle cells.^58^^,^^59^ Our use of α-SMA was intended to delineate the TM region anatomically rather than to claim TM-specific labeling. Importantly, our studies demonstrate that it can successfully deliver therapeutics to humans, as evidenced by strong mCherry expression in human TM in our ex vivo-perfusion cultured anterior segments of donor eyes.

Consistent with prior reports, wild-type MYOC is relatively short-lived with a half-life of ∼3 h.^67^ However, our observation that myocilin protein remained detectable three days after CRISPR-Cas9-mediated targeting can be explained by several factors. First, while CRISPR-Cas9 introduces indels in exon 1 and prevents new transcript production, pre-existing MYOC mRNA and protein pools are not immediately eliminated and require time to degrade. Second, unlike wild-type MYOC, mutant MYOC misfolds, accumulates in the endoplasmic reticulum, and is resistant to proteasomal degradation, resulting in slower clearance. Indeed, the same study also demonstrated that degradation of mutant MYOC occurs at a much slower rate than endogenous wild-type protein. Finally, CRISPR-Cas9 does not transfect all GTM3 cells, and non-edited cells continue to produce MYOC, further contributing to the residual protein detected. Together, these factors likely explain the persistence of myocilin protein following genome editing in our system.

Our editing strategy is intended to abolish mutant MYOC production in TM cells. While this could also reduce or eliminate endogenous MYOC, convergent animal and human genetics suggest that MYOC is not essential for TM homeostasis. Mice lacking both *Myoc* alleles exhibit no detectable ocular phenotype,^35^ and individuals with early truncation of both *MYOC* alleles have been described without apparent ocular abnormalities.^37^ Given that mutant MYOC can co-aggregate with WT myocilin, eliminating both mutant and endogenous *MYOC/Myoc* would be predicted to lower the aggregate load and ER stress, thereby improving cellular fitness. Taken together, these data support the translational premise that therapeutic MYOC ablation in the TM is unlikely to impair outflow function and may be beneficial by removing the source of proteotoxic gain-of-function.

Next-generation sequencing (NGS) of genomic DNA from pooled iridocorneal angle tissues confirmed the presence of deletions within the targeted MYOC region. Three distinct deletion patterns were detected; however, due to pooling of DNA from three eyes per sample, it was not possible to determine whether a single predominant deletion occurred across individual mice. Moreover, because the dissected tissue contained a mixture of TM, ciliary body, iris, and scleral cells, precise quantification of edited versus unedited alleles specifically within TM cells could not be obtained. These findings confirm successful on-target editing at the MYOC locus but highlight the need for future studies employing TM-specific enrichment strategies to resolve allele frequencies at the single-cell level.

Although the exact mechanism of how TM cells uptake mRNA is not clear, the phagocytic nature of TM cells likely facilitates the uptake of the liposomal mRNA, making this a targeted and efficient method for gene editing. Similar uptake of mRNA delivered via lipid nanoparticles (LNP) was observed in other phagocytic cells, including the retinal pigmented epithelium.^68^^,^^69^^,^^70^^,^^71^ While lipoplexes enabled successful delivery and initial validation of our gene editing strategy, recent advances in LNP technology have highlighted their superior efficiency, stability, and translational potential. Notably, a previous study has demonstrated the effective use of LNPs for delivering mRNA to the iridocorneal angle. In this study, LNP-mediated delivery of Cas9 mRNA achieved robust gene editing in the iridocorneal angle of the Ai9 reporter mice.^54^ Building on these advances, our laboratory is now focused on designing and optimizing LNP formulations for targeted and efficient delivery of Cas9 mRNA to the TM, with the goal of editing disease-causing *MYOC* mutations in a mouse model of glaucoma. Our current lipoplex-based studies lay the foundational groundwork for this transition and support the development of LNP-based precision gene therapies for glaucoma.

An important consideration for therapeutic translation is whether *MYOC*-targeted intervention remains effective once glaucomatous damage is already established. In the present study, CRISPR-Cas9 treatment was initiated prior to significant RGC structural loss, limiting our ability to directly address late-stage rescue. However, our previous work demonstrated that Ad5-Cas9-g*MYOC* delivery in aged Tg-*MYOC*^*Y437H*^ mice (15 months old) significantly reduced IOP,^38^ indicating that MYOC reduction can still cf. benefit even at more advanced stages of disease. Based on these findings, it is likely that *MYOC* editing initiated at later time points, such as 15 weeks post-Cre induction, when RGC functional and structural deficits are clearly observable, could protect remaining RGCs. Our future work will focus on these follow-up studies.

The CRISPR-Cas9 gene-editing tool has transformed gene-editing research by offering precise and efficient genome editing capabilities. Our study contributes to the growing body of evidence by demonstrating the effectiveness of this treatment in managing *MYOC* glaucoma. These results align with CRISPR’s success in other genetic diseases, such as sickle cell anemia and β-Thalassemia,^72^ Duchenne muscular dystrophy,^73^ and Leber Congenital Amaurosis.^74^ However, challenges such as off-target effects and immunogenicity to viral vectors remain major concerns. In this study, we demonstrated that mRNA technologies can effectively address these issues in CRISPR-based therapies. Using mRNA as a delivery platform for CRISPR-Cas9, we minimized off-target risks, while the non-viral nature of mRNA reduced immune responses, enhancing overall safety. Furthermore, mRNA-based CRISPR delivery offers additional advantages for ocular gene therapies. The eye is an immune-privileged organ, yet inflammatory responses to viral vectors remain a concern, particularly in gene-editing applications that require high or repeated dosing. By utilizing a non-viral mRNA approach, we significantly reduce the likelihood of adverse immune reactions, enhancing the safety profile of this therapeutic strategy. Additionally, mRNA can be formulated with biocompatible delivery carriers, such as lipid nanoparticles or polymer-based polyplexes, which have been extensively optimized for ocular administration. These carriers enhance cellular uptake and protect mRNA from rapid degradation, improving gene editing efficiency within TM cells.

In this study, we demonstrate proof-of-principle for mRNA delivery to the TM using liposome-based carriers. While our findings represent an important first step in optimizing non-viral gene editing approaches for the TM, several limitations of liposome-mediated mRNA delivery should be noted. The efficiency of CRISPR-Cas9 editing in GTM3 cells was modest, with T7E1 assay quantification showing ∼18% editing (Figure S7). The relatively low editing efficiency may reflect the additional steps required for mRNA-based delivery, including protein translation, as well as the need for subsequent assembly of Cas9 protein with guide RNAs, which together may slow the kinetics of genome editing. Despite this apparent limitation, functional outcomes suggest that partial editing can still produce meaningful biological effects. Western blot quantification revealed a 53% reduction in MYOC and a 51% reduction in the ER stress marker GRP78 following Cas9/gRNA treatment (Figure 3D), indicating that even incomplete editing of *MYOC* can substantially reduce pathological protein accumulation and associated stress responses. Importantly, *in vivo* experiments demonstrated that reducing, but not completely abolishing, mutant MYOC expression was sufficient to both lower IOP pressure and prevent its elevation in susceptible mice (Figures 4A and 5A). Future work will be directed toward improving the efficiency of gene editing in the TM. In particular, delivery of pre-assembled Cas9 ribonucleoprotein (RNP) complexes using advanced lipid nanoparticle formulations may circumvent some of the limitations associated with mRNA-based systems, thereby enhancing editing kinetics and efficiency. Such strategies are likely to provide more robust and consistent therapeutic outcomes in the context of *MYOC*-associated glaucoma.

Translating these findings into clinical applications is the next critical step. Viral vectors are currently the preferred method for delivering gene cargos to the TM.^75^^,^^76^ However, their use is often associated with the induction of ocular inflammation.^44^^,^^77^^,^^78^ The lipoplex-Cas9 mRNA formulation employed in our study did not induce ocular inflammation, as visualized by live OCT imaging of the anterior and posterior segments, distinguishing it from currently used viral vectors and representing a critical milestone toward clinical development. In previous work, we reported substantial off-target effects when Cas9 was delivered via lentiviral vectors.^44^ In the present study, T7 endonuclease assay and NGS revealed no detectable off-target effects, with high on-target editing of *MYOC* after lipoplex-Cas9 delivery. Together, these results validate the safety of this genome editing approach in a preclinical setting. While our results in *Tg.CreMYOC*^*Y437H*^ mice are promising; further studies are needed to validate the long-term safety, efficacy, and durability of this approach before advancing to clinical trials.

In conclusion, this study demonstrates that CRISPR-Cas9 genome editing, delivered via mRNA, holds promise as a disease-modifying therapy for *MYOC* glaucoma by targeting the entire gene, irrespective of the underlying genetic mutations. The novelty of this approach lies in its ability to circumvent the limitations of viral gene delivery while maintaining high editing efficiency and safety. As genome editing technologies continue to advance, mRNA-based CRISPR delivery has the potential to revolutionize glaucoma treatment and expand the therapeutic landscape for other inherited ocular diseases. This approach could offer a durable, safe, and effective solution for patients with *MYOC*-associated glaucoma and other genetic diseases with further optimization and validation.

## Materials and methods

### Animal husbandry

The animals were housed at the University of North Texas Health Science Center vivarium (UNTHSC) in Fort Worth, TX, USA. The animals were provided with standard chow and water ad libitum and resided in cages with dry bedding. The animals were subjected to a 12-h light-dark cycle, with lights on at 06:30 AM, and were maintained within a controlled environment featuring temperatures of 21°C–26°C and humidity levels of 40%–70%. We utilized a recently developed Cre-inducible transgenic mouse expressing the dsRed-tagged Y437H mutant of human *MYOC* (*Tg.CreMYOC*^*Y437H*^)^30^. Under baseline conditions, the mutant *MYOC* is not expressed. The activation of Cre recombinase excises the STOP cassette, allowing the expression of the dsRed-fused mutant *MYOC*. Regular PCR genotyping of these transgenic mice was performed using a forward primer (CTCAGCAGATGCTACCGTCA) and a reverse primer (TTCATGCGCTTCAAGGTGC). Both males and females were utilized in the study. The number of mice used in each experiment is denoted in the representative figures or figure legends. At the end of each experiment, mice were euthanized using CO_2_ inhalation followed by cervical dislocation.

### Antibodies and reagents

The following antibodies were used in the current study, most of which have been previously validated in published studies (see references): Myocilin monoclonal antibody (60357-1-Ig, Proteintech)^30^; RFP (600-401-379, Rockland)^79^; KDEL (NBPI-97469, Novus)^80^; ATF4 (SC-200, Santa Cruz Biotechnology)^77^; CHOP (NBP2-66856, Novus Biologicals)^77^; GRP78 (ab21685, Abcam)^81^; GRP94 (11402, Santa Cruz Biotechnology)^29^; Cas9 (632607, TaKaRa); RBPMS (118619, Gene Tex)^82^; GAPDH (3683, Cell Signaling Technology)^83^; and α-SMA (ab5694, Abcam).^77^

### mRNA-lipoplex formulation

GFP, mCherry, Cre, CleanCap Cas 9 mRNAs (TriLink BioTechnologies, San Diego, CA) were formulated by mixing them with either jetMESSENGER (*in vitro* studies) or In vivo-jetRNA (*in vivo* studies) (PolyPlus, Illkirch, France). According to the manufacturer’s protocol, each mRNA was mixed with the appropriate transfection agent at a 1:1 ratio, then combined with 30 μL of buffer (for *in vitro* studies) or 8 μL of buffer (for *in vivo* studies). After formulation, the mixture was incubated at room temperature for 15 min before use. The size distribution, PDI, and zeta potential were determined by Dynamic Light Scattering (DLS) using a Zetasizer Nano ZS (Malvern Panalytical Inc., Westborough, MA) at 25°C.

### Intracameral injections

Animals were anesthetized using 2.5% isoflurane plus 100% oxygen. Before injection, the mouse eyes were anesthetized with topical proparacaine HCl drops (0.5%) (Akorn Inc., Lake Forest, IL, USA). Mouse eyes were dilated with topical 1% cyclopentolate (Mydriacyl, Alcon Laboratories, Fort Worth, TX). A Hamilton glass micro-syringe (10 μL capacity) equipped with a 33-gauge, 1/2-inch-long needle was inserted through the cornea, approximately 2 mm anterior to the limbus, and the needle positioned parallel to the iris, aiming toward the chamber angle opposite the insertion point. mRNA-lipoplex was then slowly released into the anterior chamber over one minute, with the needle remaining in place for an additional minute before being quickly withdrawn.

### IOP measurements

IOPs were measured under gas anesthesia (isoflurane at 2.5%; oxygen at 0.8 L/min). A TonoLab tonometer (Colonial Medical Supply, Londonderry, NH, USA) was used for IOP measurements as previously described.^77^^,^^83^ IOP measurements were conducted weekly in a masked fashion at the same time (between 8 and 10 AM). An average of seven readings was taken during each session. Care was taken to align the tonometer tip perpendicular to the central cornea while obtaining the measurements. To minimize the influence of isoflurane on IOP, all measurements were performed immediately when the lid reflex was lost and within a 5-min window.

### Aqueous humor outflow facility

Aqueous humor outflow facilities were measured 20 weeks after injection. The outflow facilities in both vehicle and cre-mRNA lipoplex-injected eyes were measured using the constant-flow infusion technique, as previously described.^77^^,^^82^ Briefly, mice were anesthetized using a ketamine/xylazine (100/10 mg/kg) and maintained at 37°C using a heating pad. Topical proparacaine HCl eye drops were used for corneal anesthesia. Cannulation of the anterior chambers was performed with 30-gauge needles inserted through the cornea, ensuring no contact with surrounding eye structures. These needles were connected to a pressure transducer within a perfusion system. The system was filled with sterile phosphate buffer solution (PBS), and eyes were infused at flow rates ranging from 0.1 μL/min to 0.5 μL/min (in 0.1 μL/min increments). Stabilized pressures were recorded at these flow intervals, and the mean stabilized pressure was calculated. The aqueous humor outflow facility was then determined from the slope of a plot correlating the mean stabilized pressure with the flow rate. Saline drops were applied to prevent corneal drying throughout the procedure.

### Pattern electroretinography

Pattern electroretinography (pERG) was conducted to assess RGC function using the binocular pERG system (Jorvec, Miami, FL), as described previously.^82^ Briefly, after overnight dark adaptation, mice were anesthetized with a ketamine/xylazine solution (100/10 mg/kg) and positioned on a heated stage for the duration of the procedure. PERG measurements and analyses were performed according to the manufacturer’s instructions.

### Fundus imaging and optical coherence tomography

Imaging and analyses of the retinal thickness were performed using the Heidelberg Eye Explorer 1.10.4.0 spectral-domain OCT (Heidelberg Engineering GmbH, Heidelberg, Germany). Mice were positioned on a heated stage, and OCT imaging was conducted under gas anesthesia (isoflurane at 2.5%; oxygen at 0.8 L/min). Anterior chamber angle imaging was performed using the Spectralis anterior segment module. After anterior segment imaging, one drop of 1% tropicamide (1% Mydriacyl, Alcon, Fort Worth, TX, USA) was applied for mydriasis to obtain high-quality retinal images. The outer retinal thickness was defined as the thickness of retinal tissue between the external limiting membrane and Bruch’s membrane.

### Whole-mount retina staining

To assess the total number of surviving RGCs in both vehicle and cre-mRNA lipoplex-injected eyes, whole-mount retina staining with RNA-binding protein with multiple splicing (RBPMS) antibody (catalog # 118619, Gene Tex) was performed as described previously.^82^ Upon euthanizing the mice, eyes were enucleated and fixed in 4% paraformaldehyde (PFA) for 12 h at 4°C. Following a PBS rinse, the anterior chamber was removed, and the retinas were carefully separated from the posterior cup. Isolated retinas were immersed in blocking buffer (10% goat serum and 0.2% Triton X-100 in 1x PBS) for 12 h at 4°C. Subsequently, the retinas were incubated with RBPMS antibody (1:400 dilution) for 3 days at 4°C, followed by a 2-h PBS wash. After washing, retinas were treated with a corresponding secondary antibody (goat anti-rabbit 568, 1:500; Invitrogen) for 2 h at room temperature and then washed for 1 h in PBS. Finally, the retinas were mounted using a medium containing DAPI nuclear stain. To count RGCs, a minimum of 16 non-overlapping images covering the entire retina were captured at 200× magnification using a Keyence fluorescence microscope (Itasca, IL, USA). We evaluated equivalent eccentricities for each area identified as inner, middle, and peripheral retina. RBPMS-positive cells were quantified using ImageJ software.^84^

### Assessment of optic nerve damage by PPD staining

P-phenylenediamine (PPD) staining was used to examine optic nerve degeneration in Veh (Control) and cre-mRNA-injected mice, as described previously.^85^ PPD selectively and lightly stains the myelin sheath of healthy axons, while the axoplasm of damaged or dying axons appears dark. In brief, optic nerves were isolated from the eyeball and fixed overnight in a phosphate-buffered 3% glutaraldehyde/paraformaldehyde mixture at 4°C. Subsequently, the optic nerves were treated overnight with 1% osmium tetroxide at 4°C, followed by rinsing in 0.1 M phosphate buffer and 0.1 M sodium-acetate buffer, and dehydration in graded ethanol concentrations. After embedding in resin (Eponate-12; Ted Pella), 1 μm sections were prepared and stained in 1% PPD for 10 min. Axons in the optic nerve were manually counted using a previously described method.^27^ Specifically, 10 non-overlapping optic nerve images were captured at 1630× magnification, and axons were counted within an area equal to 10% of the total nerve cross-sectional area.

### Histology and immunostaining

Mice were euthanized, and their eyes were carefully enucleated. The eyes were fixed overnight at 4°C in 4% PFA (Electron Microscopy Sciences, Hatfield, PA, USA). The following day, the eyes were washed with 1x PBS (Sigma-Aldrich) and cryopreserved using a graded sucrose series (10% and 20%) overnight for each stage. They were then embedded in an OCT compound and sectioned at a thickness of 10 μm using a cryostat (Leica Biosystems, Inc., Buffalo Grove, IL, USA). The sections were allowed to air-dry at room temperature before further processing. For immunostaining, the sections were permeabilized in 0.1% Triton X-100 in 1X PBS for 15 min and then blocked with 10% normal goat serum (EMD Millipore Corp) in 0.2% Triton X-100 (in PBS; Fisher BioReagents, Fair Lawn, NJ, USA) for 2 h. Slides were incubated overnight with the primary antibody (1:100), washed three times in PBS, and then incubated for 2 h with Alexa Fluor secondary antibodies (1:200; Invitrogen). Sections were mounted with a DAPI-mounting solution, and the general morphology of the anterior segment, including the TM and corneal structure, was assessed via light microscopy. Images were acquired using a Keyence fluorescence microscope (Itasca, IL, USA) as described previously.^80^

For *in vitro* studies, TM cells were cultured in 8-well chamber slides (Lab-Tek Nunc Brand Products, Rochester, NY, USA) and fixed with 4% PFA for 20 min, followed by PBS washes. Fixed cells were blocked with 10% goat serum in 0.1% Triton X-100 for 2 h. Primary antibodies against MYOC (Catalog #60357, Proteintech) and KDEL (Catalog #NBP1-97469, Novus) were incubated overnight, followed by four washes with 1x PBS. Alexa Fluor secondary antibodies (1:500; Invitrogen, Life Technologies, Grand Island, NY, USA) were incubated at room temperature for 2 h. After washing, slides were mounted with DAPI as described previously.^81^^,^^86^

### Design of human *MYOC*-specific gRNA

The guide RNA (gRNA) Hs.Cas9.*MYOC*.1.AA was specifically designed (Integrated DNA Technologies, Inc., Coralville, Iowa, USA) to target the *MYOC* gene at the genomic location NC_000001.11: 171652378-171652359. The protospacer sequence, CAGCACCCAACGCTTAGACC, was selected to maximize targeting efficiency and specificity. Forward (CTTGCCTTAGTCGCTTCTTCT) and reverse (TGTTCTGGCTGGCTGTTATT) PCR primers were used to amplify a 1014 bp region surrounding the target site (Position 310 of transcript NM_000261.2 (Exon 1)).

### Cell culture and *in vitro* transfection

Immortalized GTM3 cells were transfected using the lipofectamine 3000 transfection kit (Invitrogen, Life Technologies, Grand Island, NY, USA) with pDsRed2-*MYOC* plasmids, which express either wild-type (WT) or mutant *MYOC* (Y437H or G364V) tagged with dsRed at the C-terminus. Following transfection, GTM3 cells were allowed to reach confluence and then treated with G418 antibiotic at a concentration of 0.6 mg/mL (Gibco, Life Technologies, Grand Island, NY, USA) for 7–10 days to select for stably transfected colonies. Individual colonies were subsequently selected and expanded. The stable cell lines expressing dsRed-tagged *MYOC* (with or without mutations) were characterized as previously described.^81^ These cell lines were maintained in DMEM (Sigma-Aldrich Corp, St. Louis, MO, USA) supplemented with G418, 10% FBS (Gibco), and streptomycin (Gibco). For non-viral transfection, GTM3 cells stably expressing a mutant *MYOC* were initially plated at 40% confluency and then incubated with a lipoplex formulation containing mRNA that expressed Cas9 and a gRNA targeting *MYOC*, or with a non-targeting control gRNA. On day 3 post-treatment, after the cells had reached confluence, they were processed for DNA isolation, western blotting, and immunostaining.

Skin fibroblasts from *Tg.CreMYOC*^*Y437H*^ mice were cultured and maintained in high-glucose Dulbecco’s Minimal Essential Medium (DMEM) supplemented with 10% FBS, 100 U/mL penicillin, and 100 U/mL streptomycin. Cells were grown in a 6-well plate and transfected with a mixture of Cre mRNA (TriLink BioTechnologies, San Diego, CA) and jetMESSENGER transfecting agent (PolyPlus, Illkirch-France) in a one-to-one ratio, gently mixing it into the medium.

### Western blot analysis

GTM3 cells or iridocorneal rings containing the TM were lysed in RIPA buffer (Thermo Fisher Scientific). Equal amounts of protein (20–30 μg) were loaded per lane, separated on denaturing 4%–12% NuPAGE Bis-Tris gradient gels (Invitrogen), and transferred onto PVDF membranes (MilliporeSigma). Membranes were blocked with 10% nonfat dried milk for 1 h at room temperature, followed by overnight incubation at 4°C with specific primary antibodies on a rotating shaker. After three washes with PBST, membranes were incubated with HRP-conjugated secondary antibodies for 90 min. Protein signals were visualized using ECL detection reagents (SuperSignal West Femto Maximum Sensitivity Substrate; Invitrogen) and imaged with the LI-COR Odyssey Fc system. To confirm equal protein loading, the same blots were re-probed with a GAPDH antibody (Cell Signaling Technology).

### Perfusion cultured anterior segment model

Human donor eyes were obtained from the Willed Body Program at UNTHSC (Fort Worth, TX) in accordance with the Declaration of Helsinki guidelines. The anterior segments of paired eyes were prepared for *ex vivo* perfusion culture as previously described.^38^ A bolus of 5 μL mCherry lipoplex (1 μg/μL) was injected into the anterior segment of one eye, while the contralateral eye received a control injection. After injection, the eyes were perfused at a constant flow rate of 2.5 μL/min. Following perfusion for 4 days, the TM was carefully dissected, stained with DAPI, and imaged.

### Genomic endonuclease assay

GTM3 cells were treated with lipoplex containing mRNA Cas9 plus g*MYOC*, a scrambled gRNA, or an mRNA Cas9 with no gRNA, and genomic DNA was isolated after 48 h. The extraction was performed using the NucleoSpin Tissue kit (catalog# 740952, USA). To specifically amplify the *MYOC* gene, which is the target of the selected gRNA, a polymerase chain reaction (PCR) was conducted. Following amplification, the PCR products underwent a denaturation and reannealing process, adhering to the protocol provided by the Alt-R Genome Editing Detection Kit (catalog# 1075932, Integrated DNA Technologies, USA). This step facilitated the formation of mismatched heteroduplex DNA, comprising strands that exhibited Cas9-induced insertions or deletions (indels) paired with either wild-type strands or strands containing different indels. These heteroduplexes were detected using T7 endonuclease, an enzyme that specifically recognizes and cleaves mismatched regions within the DNA. T7E1 produced distinct cleaved products that were further analyzed by targeting the heteroduplexes. The resulting fragments from this cleavage were then subjected to gel electrophoresis.

### Next-generation sequencing analysis

Genomic DNA was isolated from TM tissue collected from *Tg.CreMYOC*^*Y437H*^ mice following gene editing. DNA extraction was performed using the NucleoSpin Tissue kit (catalog# 740952, USA) according to the manufacturer’s instructions. A 385 bp region of the *MYOC* gene was amplified by PCR using the primers *MYOC*-NGS-FW (5′-AGATGCCAGCTGTCCAGCT-3′) and *MYOC*-NGS-REV (5′-TAGGCAGTCTCCAACTCTCTGGTT-3′). PCR products were purified using the Invitrogen PCR Purification Kit (Cat. no. K310001). Amplicon sequencing and subsequent data analysis were performed by Azenta Life Sciences. The presence of gene editing in Cas9mRNA-*MYOC* sgRNA-treated samples was confirmed by aligning the sequencing data to the *MYOC* reference sequence.

### The study approval

All animal studies were conducted in accordance with the guidelines and regulations established by the UNTHSC Institutional Animal Care and Use Committee (IACUC) and the ARVO Statement for the Use of Animals in Ophthalmic and Vision Research.

### Statistics

For all experiments, “n” refers to the number of eyes. GraphPad Prism 9.0 was used to generate graphs and run statistical analyses (GraphPad, San Diego, CA, USA). Significance was determined at a threshold of *p* < 0.05. Data are presented as mean ± SEM. For comparisons between the two groups, an unpaired Student’s t-test (two-tailed) was utilized. For analyses involving more than two groups, such as the IOP results, repeated-measures two-way ANOVA was employed, followed by a Bonferroni post-hoc correction.

## Data and code availability

The datasets generated and/or analyzed during the current study are available from the corresponding author upon reasonable request.

## Acknowledgments

These studies were supported by the 10.13039/100000002National Institutes of Health (EY034333 and EY026177), Alcon Research Foundation, and 10.13039/100001818Research to Prevent Blindness. The authors acknowledge support from 10.13039/100000002NIH grant EY034238 and an unrestricted grant from 10.13039/100001818Research to Prevent Blindness to the Gavin Herbert Eye Institute at the 10.13039/100008476University of California, Irvine.

## Author contributions

G.Z. conceptualized and designed the research studies. S.Y. and B.R.K. performed experiments and analyzed data. S.R., L.L., R.K., and P.M. assisted in some experiments. S.Y. and G.Z. wrote the manuscript. A.F.C. provided resources and assisted in the manuscript editing. All authors discussed the results and implications and commented on the manuscript at all stages.

## Declaration of interests

The authors declare no competing interests.

## Supplemental information

Document S1. Figures S1–S9

Document S2. Article plus supplemental information
