# Supplementary material for: Therapeutic mRNA delivery of CRISPR-Cas9 to the trabecular meshwork reverses ocular hypertension in myocilin glaucoma
Source: Mol Ther Methods Clin Dev. 2025 Oct 11;33(4):101614. doi: 10.1016/j.omtm.2025.101614 (PMC12594927; doi:10.1016/j.omtm.2025.101614)
Supplement: Document S1. Figures S1–S9 [file mmc1.pdf]

OMTM, Volume *33*

Supplemental information

Therapeutic mRNA delivery of CRISPR-Cas9 to the trabecular meshwork reverses ocular hypertension in myocilin glaucoma

Sam Yacoub, Balasankara Reddy Kaipa, Linya Li, Sarahi Rios, Ramesh Kasetti, Prabhavathi Maddineni, Abbot F. Clark, and Gulab S. Zode

Supplemental Information
Yacoub et al.

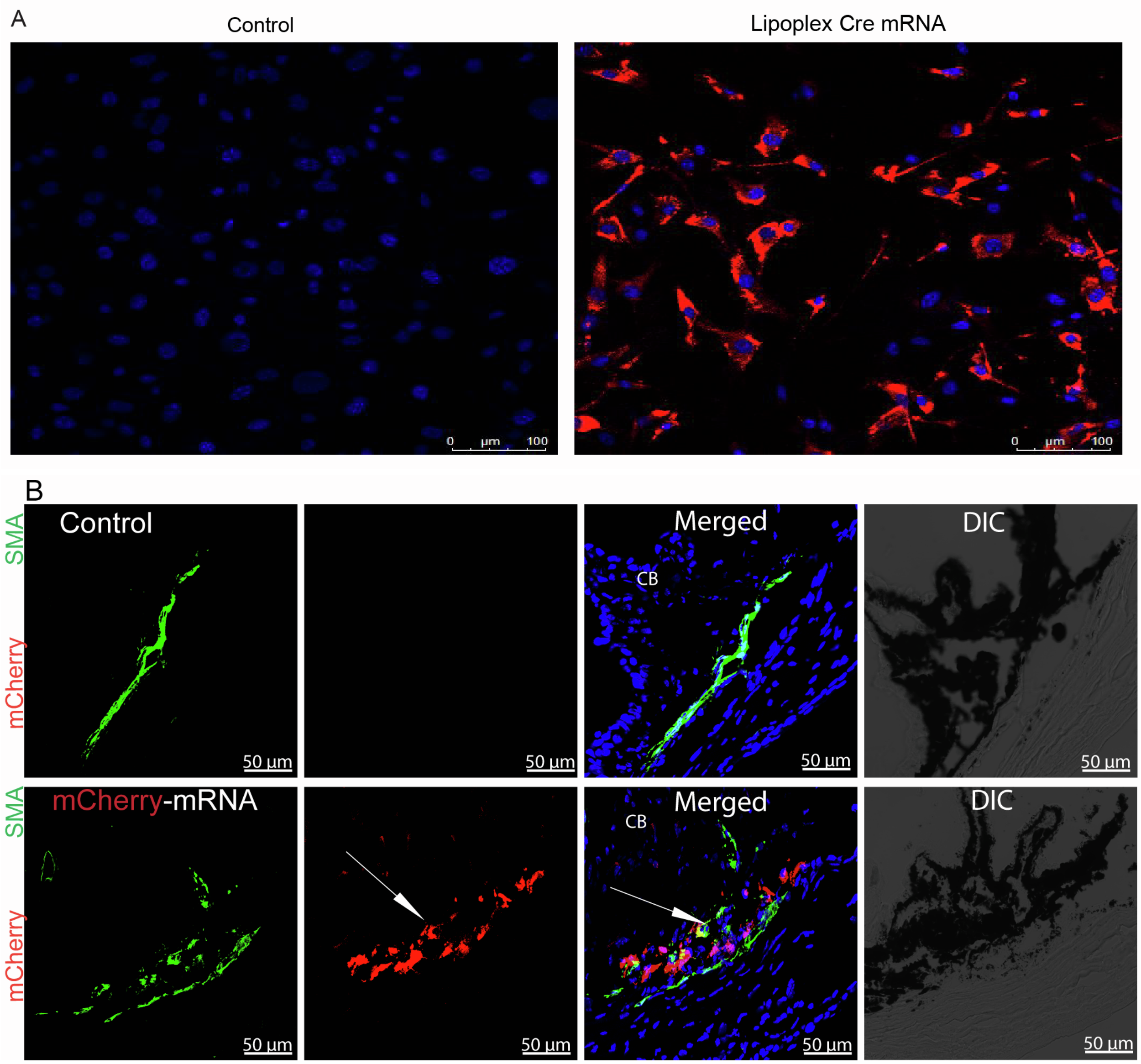

Figure S1. A) Lipoplex formulated with Cre-mRNA demonstrates Cre functional activity in cultured skin fibroblasts. Cultured skin fibroblasts from *Tg.CreMYOC*^{Y437H} mice were incubated with lipoplex carrying Cre mRNA. Expression of DsRed was visualized after 24 hours of treatment. Under normal conditions, these fibroblasts do not express DsRed-fused mutant MYOC (Control). Most skin fibroblasts treated with Cre-mRNA induced DsRed expression (blue = DAPI); n=3.

B) mCherry protein localization in mouse TM: Lipoplex-mCherry mRNA was injected intracamerally, and anterior segment sections were examined for mCherry protein expression. To further confirm the localization, sections were co-stained with α-smooth

muscle actin (α-SMA), which labels both TM and ciliary muscle cells. Separate channels of α-SMA (green), mCherry (red), and merged images with DAPI and DIC are shown. Robust mCherry fluorescence was detected in the TM region, indicating efficient lipoplex-mediated mRNA transfection in TM cells in vivo. Low-magnification merged images are shown in Figure 1D, whereas higher-magnification images with separate channels are presented here.

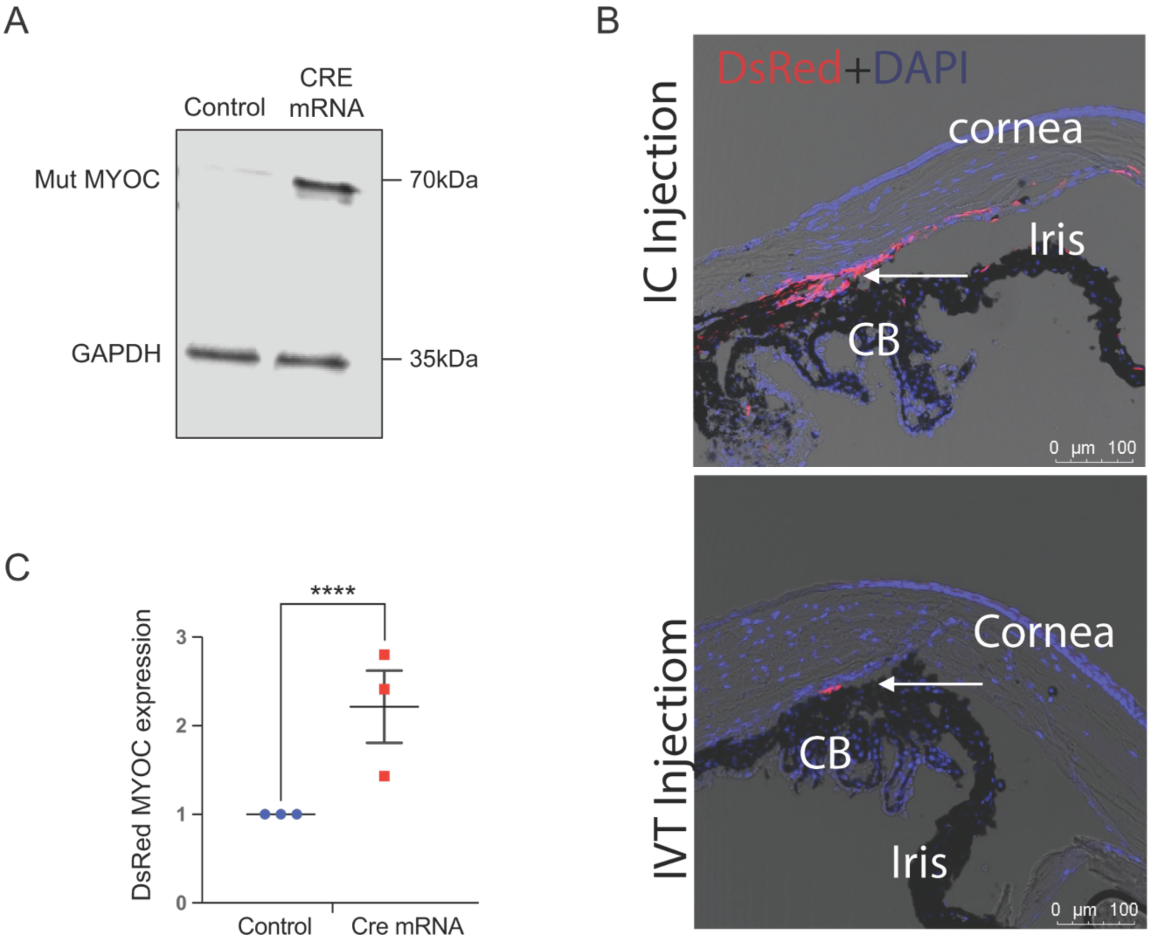

Figure S2. IC injection of lipoplex carrying Cre mRNA induces mutant MYOC protein in *Tg.CreMYOC*^{Y437H} mice and demonstrates Cre functional activity in the TM. A) Western blot analysis of iridocorneal angle tissues using RFP antibody shows the presence of human mutant myocilin in lipoplex Cre mRNA-injected eyes compared to control. Note that endogenous MYOC was not detected since we have utilized the RFP antibody, which only recognizes DsRed protein fused to MYOC. **B)** *Tg.CreMYOC*^{Y437H} mice were injected intracamerally (IC) or intravitreally (IVT) with lipoplex carrying Cre mRNA, and functional Cre activity was examined in the anterior segment cross-sections. Cre-mRNA-injected via the IC route demonstrated a robust DsRed expression in the TM of *Tg.CreMYOC*^{Y437H} mice. In addition, we observed some sporadic DsRed expression in

the corneal endothelium. We observed lower DsRed expression in the TM via the IVT route. The IC route produced more consistent and robust expression in the TM. Based on these results, we selected IC injection for all subsequent studies. Arrows indicate TM. N=3. **C)** DsRed fluorescence analysis showing significant induction of DsRed fluorescence in the TM of Cre mRNA-injected *Tg.CreMYOC*^{Y437H} mice compared to the controls. N=3.

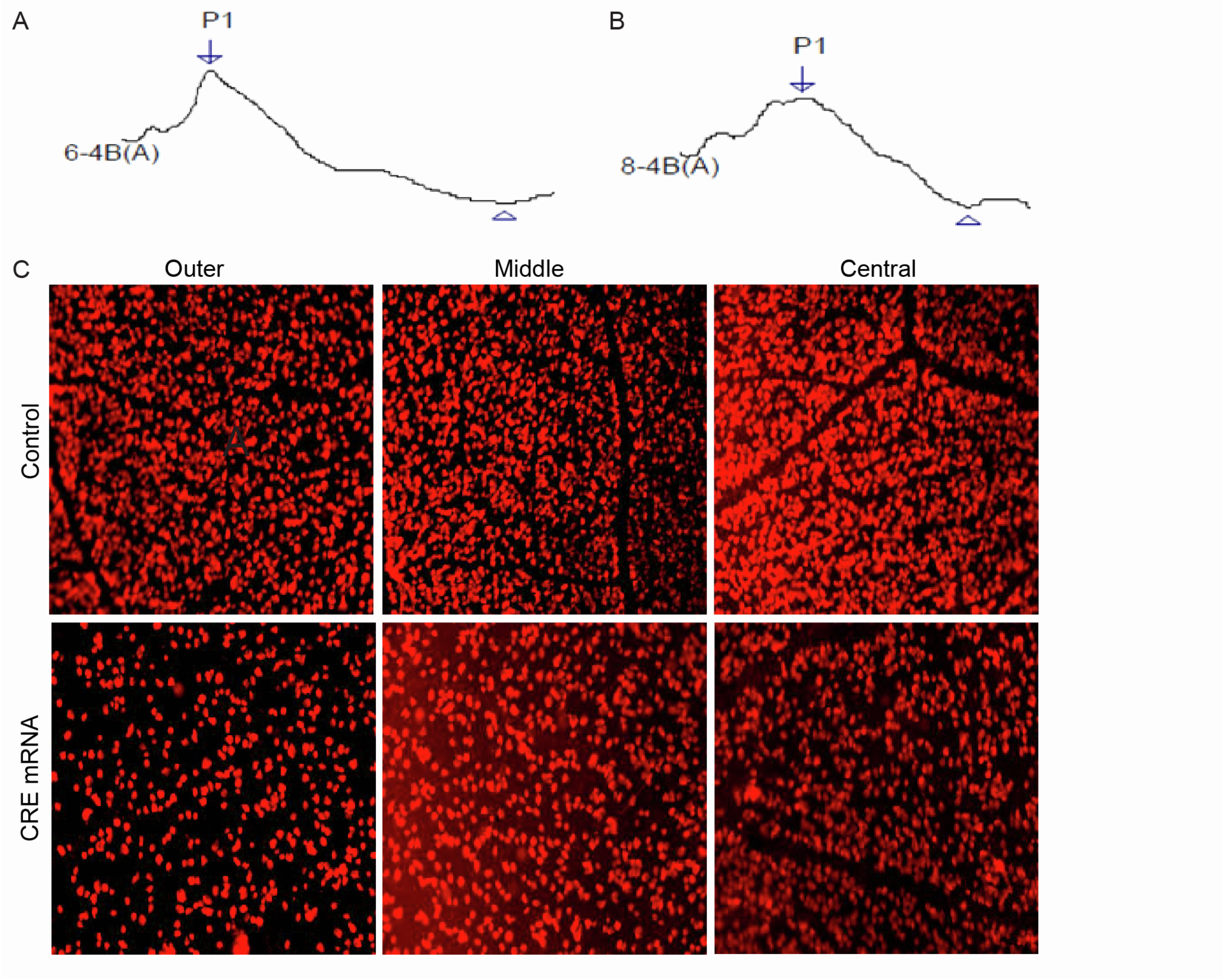

Figure S3: Sustained IOP elevation induces RGC functional and structural loss. RGC loss and optic nerve degeneration were examined in ocular hypertensive Cre mRNA-injected *Tg.CreMYOC*^{Y437H} mice. **A, B)** Representative of PERG demonstrating

significantly reduced PERG amplitude (B = control; C = Cre-mRNA). **(C)** Representative RBPMS staining of whole-mount retinas demonstrating structural loss of RGCs in *Tg.CreMYOC*^{Y437H} mice 15 weeks after Cre-mRNA injection, particularly in the peripheral retina.

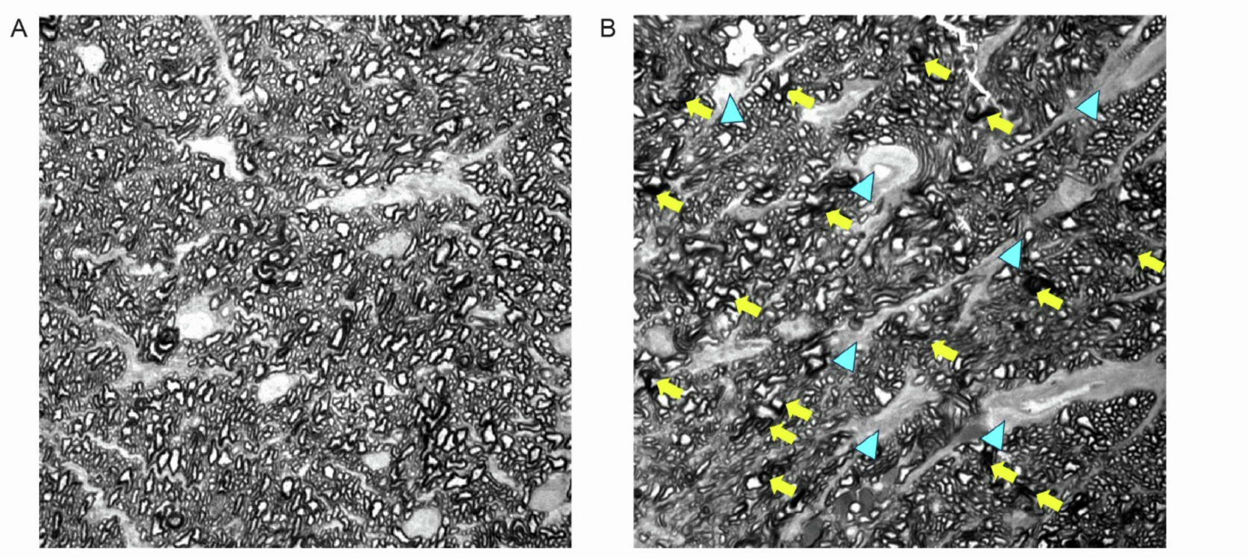

Figure S4: Sustained IOP elevation induces optic nerve degeneration. RGC loss and optic nerve degeneration were examined in ocular hypertensive Cre mRNA-injected *Tg.CreMYOC*^{Y437H} mice. (**D, E**) Representative images of PPD-stained optic nerves showing mild axonal degeneration as evident from darkly stained axons (yellow arrow) and the presence of glial scar formation (blue arrow) in Cre-injected *Tg.CreMYOC*^{Y437H} mice (D = control; E = Cre-mRNA).

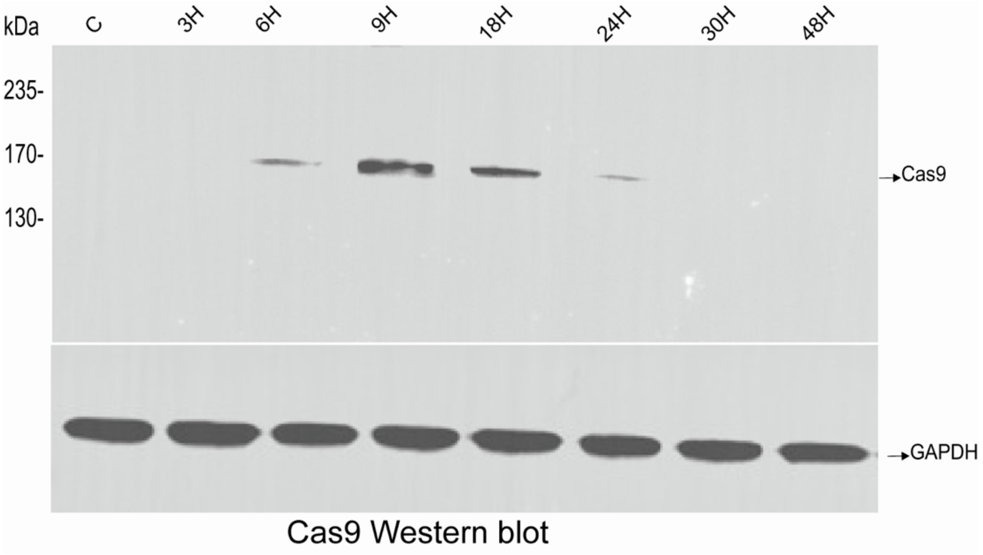

Figure S5: Time course of Cas9 protein expression following lipoplex mRNA transfection in GTM3 cells. GTM3 cells were transfected with lipoplex-formulated Cas9 mRNA and harvested at multiple time points (3, 6, 9, 18, 24, 30, and 48 hours) post-transfection. Western blot analysis was performed using an anti-Cas9 antibody, with GAPDH serving as a loading control. Cas9 protein expression was first detectable at 6 hours, peaked between 7–9 hours, and gradually declined thereafter. By 24–30 hours, only low levels of Cas9 were detected, and expression was nearly absent by 48 hours. Control (untreated) cells did not show Cas9 expression. These results confirm that lipoplex-mediated delivery of Cas9 mRNA leads to rapid, transient expression of Cas9 protein in GTM3 cells. The expression kinetics are consistent with the expected short-lived translation profile of exogenous mRNA, supporting the rationale for transient delivery to minimize prolonged Cas9 exposure and reduce the risk of off-target genome editing.

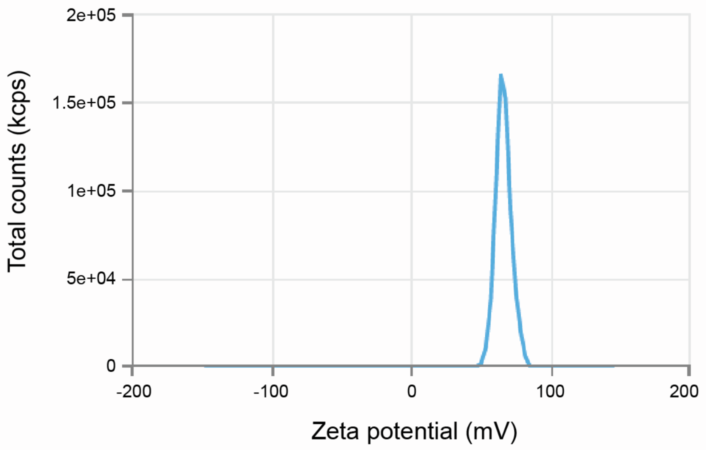

Figure S6: Peak analysis of zeta potential and particle size distribution reveals that lipoplex mRNA exhibits a uniform particle size with >95% distribution.

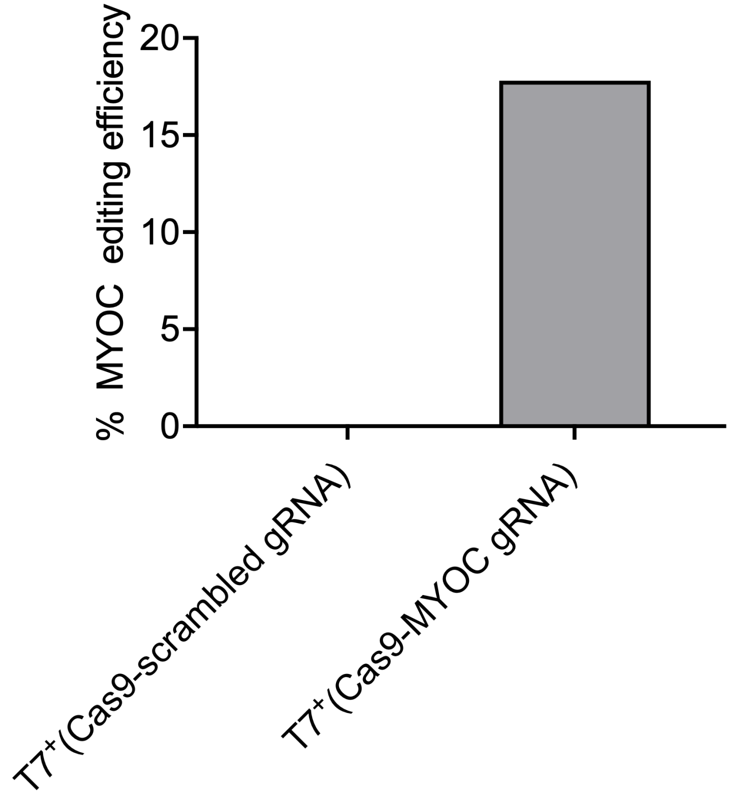

Figure S7: T7 endonuclease assay demonstrating MYOC gene editing efficiency in GTM3 cells. Genomic DNA isolated from GTM3 cells transfected with Cas9 and MYOC-targeting gRNA was subjected to PCR amplification of the target locus, followed by T7 endonuclease I digestion. Cleaved and uncleaved bands were visualized on agarose gels (shown in Figure 4B), and band intensities were quantified using ImageJ. Editing efficiency was calculated as the ratio of cleaved to uncleaved band intensity. Cas9/gRNA transfection produced detectable cleavage products, confirming targeted editing of the MYOC locus. Quantification of band intensities using ImageJ revealed a significant increase (18%) in the cut/uncut ratio in Cas9-treated samples compared to controls, indicating successful genome editing at the MYOC locus.

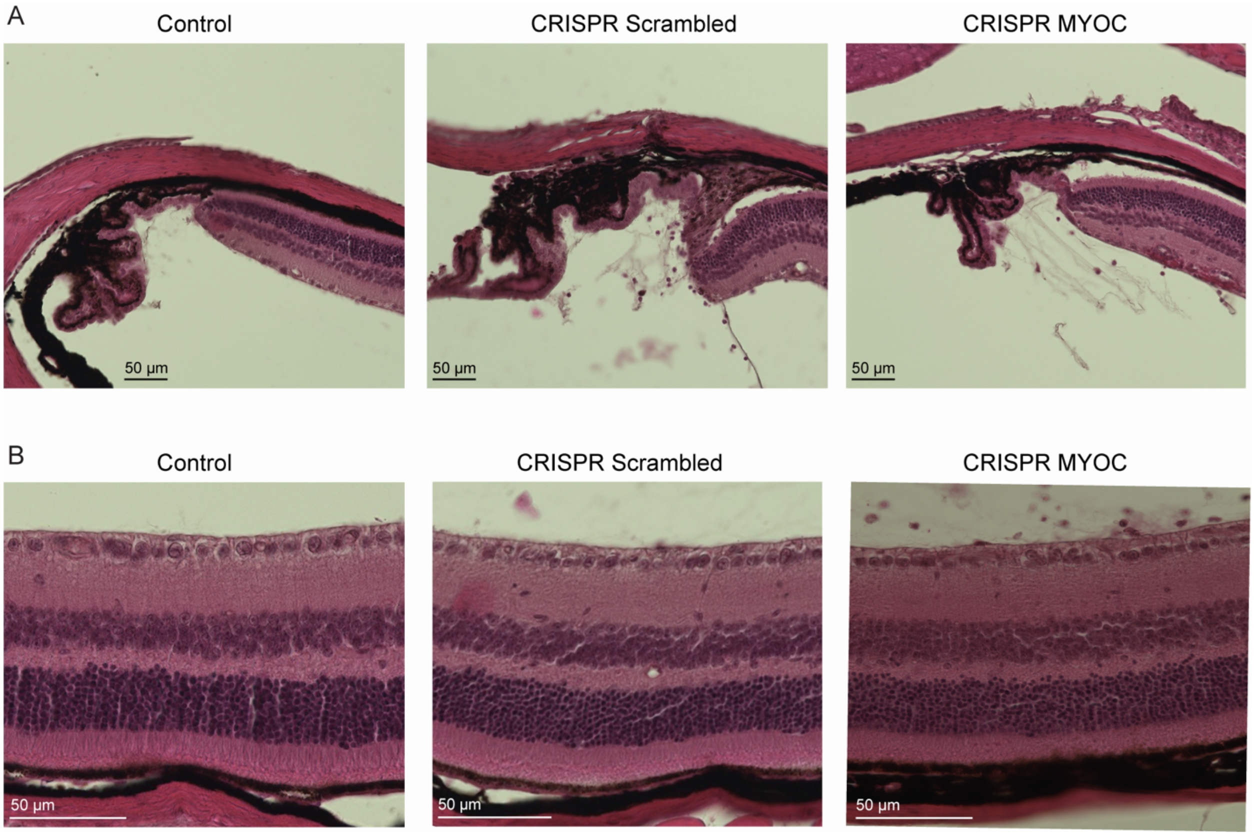

Figure S8: Representative H & E images showing largely preserved **A)** anterior and **B)** posterior segment structures in control and Cas9 mRNA with scrambled or *MYOC* gRNA injected *Tg.CreMYOC*^{Y437H} mice. No gross abnormalities or signs of ocular inflammation were observed across groups. N=3.

Sequence	PAM	Score	#MM	Gene	Locus	
AAGC-CACAAAGCTTAGACC	AGG	15	4		chr18:-76319740	
CAGTGCC-AACGCTCAGACC	CAG	52	4	GM32611	chr4:-125200682	
CACCACACAGCGCCTAGACC	CAG	60	4	NFKBIA	chr12:-55489784	
GAGCACCCGAC-CTTAGGCC	CAG	71	4	DDN	chr15:-98805686	
CAACACAAAACGCTTTGACC	AGG	73	4	LAMA5	chr2:-180180126	
CAGCACCGATC-CTCAGACC	TAG	81	4	GM8273	chr4:+8648319	

Figure S9: *In silico* analysis by Cas9 finder revealed a low likelihood of off-target activity.
